# Identification of circular RNAs regulating cardiomyocyte proliferation in neonatal pig hearts

**DOI:** 10.1172/jci.insight.175625

**Published:** 2024-06-25

**Authors:** Ling Tang, Verah Nyarige, Pengsheng Li, Junwen Wang, Wuqiang Zhu

**Affiliations:** 1 Departments of Cardiovascular Medicine and Physiology and Biomedical Engineering, Center for Regenerative Biotherapeutics, and; 2 Department of Quantitative Health Sciences Research, Center for Individualized Medicine, Mayo Clinic Arizona, Scottsdale, Arizona, USA.; 3 Division of Applied Oral Sciences and Community Dental Care, Faculty of Dentistry, the University of Hong Kong, Hong Kong SAR, China.

**Keywords:** Cardiology, Cardiovascular disease

## Abstract

Little is known about the expression patterns and functions of circular RNAs (circRNAs) in the heart of large mammals. In this study, we examined the expression profiles of circRNAs, microRNAs (miRNAs), and messenger RNAs (mRNAs) in neonatal pig hearts. Pig heart samples collected on postnatal days 1 (P1), 3 (P3), 7 (P7), and 28 (P28) were sent for total RNA sequencing. Our data revealed a total of 7,000 circRNAs in the 24 pig hearts. Pathway enrichment analysis of hallmark gene sets demonstrated that differentially expressed circRNAs were engaged in different pathways. The most significant difference was observed between P1 and the other 3 groups (P3, P7, and P28) in pathways related to cell cycle and muscle development. Out of the 10 circRNAs that were validated through real-time quantitative PCR to verify their expression, 6 exhibited significant effects on cell cycle activity in human induced pluripotent stem cell–derived cardiomyocytes following small interfering RNA–mediated knockdown. circRNA-miRNA-mRNA networks were constructed to understand the potential mechanisms of circRNAs in the heart. In conclusion, our study provided a data set for exploring the roles of circRNAs in pig hearts. In addition, we identified several circRNAs that regulate cardiomyocyte cell cycle.

## Abstract 2

Expression profiles of mRNA, miRNA, and circRNA in neonatal pig hearts suggest potential roles for circRNAs in cardiac development.

## Introduction

Heart failure continues to be a major cause of death worldwide. The primary reason for heart failure is the degeneration or loss of functional cardiomyocytes after injury. It is well established that cardiomyocytes in adult mammals possess limited regenerative potential because of cell cycle exit. Therefore, myocardium loss after myocardial injuries is typically replaced by fibrotic scar. Thus, replenishing lost cardiomyocytes emerges as a promising strategy for repairing damaged myocardium and improving heart function in preclinical models of heart failure. It was reported that the heart of neonatal mice retains regenerative capacity within the first week after birth, which is mediated by proliferation of endogenous cardiomyocytes (1). Several cell types and pathways are involved in cardiomyocyte proliferation, which includes hormones, revascularization, nerve signaling, immune response, extracellular matrix, metabolism, and gene expressions (2). We have recently shown that neonatal pigs can also regenerate lost myocardium in the first 2 days after birth in response to myocardial injuries (3). Specifically, hearts of postnatal day 1 (P1) pigs hold robust regeneration capacity following myocardial infarction. This regeneration is mediated by the proliferation of preexisting cardiomyocytes, which is lost when cardiomyocytes permanently exit the cell cycle. Thus far, molecular mechanisms underlying the injury-induced activation of cardiomyocyte cell cycle in neonatal pig hearts remain largely unknown.

Circular RNA (circRNA) is a type of RNA molecule that forms a closed-loop structure. circRNAs are divided into 4 categories: ecRNAs (exonic circRNAs), which are derived from single or multiple exons; ciRNAs (circular intronic RNAs), which are derived from introns; EIciRNAs (exon-intron circRNAs), which are composed of exons and introns; and tricRNAs (tRNA intronic circRNAs), which are formed by splicing tRNA introns (4). Previous studies have reported consistent expression of circRNAs in various human tissues and bodily fluids, including blood, saliva, and urine (5–7). circRNAs play an important role in regulating various biological processes. The unique closed-loop structure renders them resistant to degradation by ribonucleases and ensures stability. This stability allows circRNAs to accumulate in cells, making them potential biomarkers for various cardiovascular conditions. circRNAs carry out their functions through mechanisms related to transcription regulation, such as miRNA sponging, formation of RNA-protein complexes, and interactions with RNA-binding proteins (4, 8). circRNAs can act as microRNA sponges, binding to and sequestering miRNAs, thus preventing them from binding to their target mRNAs and suppressing gene expression. Additionally, circRNAs are well known for their specificity to certain tissues and developmental stages (8).

Recent studies highlighted the importance of circRNAs in the regulation of cardiovascular functions (9). The involvement of circRNAs in regulating cardiomyocyte proliferation and heart regeneration has been established for only a few circRNAs including circNfix (10), circHipk3 (11), circCDYL (12), and circMdc1 (13). However, more research has been done to identify new circRNAs that are involved in regulating the pathophysiology of the diseased heart, such as cardiac inflammation (14–16), apoptosis (17–32), autophagy (33–35), ferroptosis (36), angiogenesis (37, 38), and fibrosis (39), and modulating the expression and/or activity of these circRNAs has been shown to provide cardioprotection following myocardial injuries.

Despite these encouraging results, the majority of these studies were performed on rodents. The expression patterns and function of circRNAs in the heart of large animals are essentially unknown. The main objective of this study is to fill this knowledge gap by examining the expression profiles and functions of circRNAs in neonatal pig hearts. We were particularly interested in identifying the circRNAs that regulate cardiomyocyte cell cycle and proliferation. Toward this goal, we performed total RNA sequencing with pig heart samples collected on P1, P3, P7, and P28 and analyzed the expression profiles of circRNAs, miRNAs, and mRNAs. Our data revealed significant changes in the expression of circRNAs at different postnatal stages (i.e., from P1 to P28), and pathway enrichment analysis highlighted circRNAs that are associated with cell division and proliferation. Functional analysis verified the cell cycle function of 6 new circRNAs in cardiomyocytes. By predicting interactions between circRNAs, miRNAs, and mRNAs, we were able to infer potential relationships and construct an integrated regulatory network. This comprehensive analysis provided insights into the complex regulatory processes involving circRNAs, miRNAs, and mRNAs during postnatal heart development in newborn pigs. Additionally, our study generated a data set for further exploration of circRNA roles in the heart of neonatal pigs.

## Results

We performed RNA sequencing of mRNAs and noncoding RNAs, including circRNAs and miRNAs, using samples from neonatal pig hearts at different developmental stages (P1, P3, P7, and P28, with 6 animals at each age). The heart samples were sent to 2 companies for sequencing (Supplemental Table 1; supplemental material available online with this article; https://doi.org/10.1172/jci.insight.175625DS1). In the first batch, 12 samples from animals of different ages (3 animals at each age) were sent to Novogene for circRNA sequencing (annotated as “nv1”). A second batch of 12 more samples was sent to CD Genomics (annotated as “cd”) for total RNA sequencing. In the third batch, new samples from the same 12 animals in the first batch were sent to Novogene (annotated as “nv2”) for total RNA sequencing again. We validated the sequencing data using real-time quantitative PCR (qRT-PCR), performed differential expression analysis and pathway enrichment analysis using different bioinformatics tools, and conducted functional experiments in the cultured cardiomyocytes via siRNA-mediated knockdown of selected circRNA candidates. Schematics of experimental design are shown in Supplemental Figure 1.

### Differential expression analysis of circRNAs in neonatal pig hearts.

We analyzed circRNA expression profiles in neonatal pig hearts using circRNA-sequencing data from all 3 batches (nv1, cd, and nv2; 36 samples), which covered a total of about 7,000 circRNAs. A principal component analysis (PCA) of our batch-corrected samples exhibited a great random mixing of samples from the 3 batches, implying successful cross-sample batch correction. The most noteworthy difference was observed between P1 and the other 3 groups. Although samples within the other groups exhibited closer similarities to one another, there were occasional individual samples that displayed similarities to samples from different groups. This occurrence is likely due to the deliberate selection of samples from neonatal pigs to detect subtle differences in their cardiac regenerative function. Therefore, the experimental design remains reasonable, and the biological replications are considered satisfactory (Supplemental Figure 2A).

**Figure 1:**
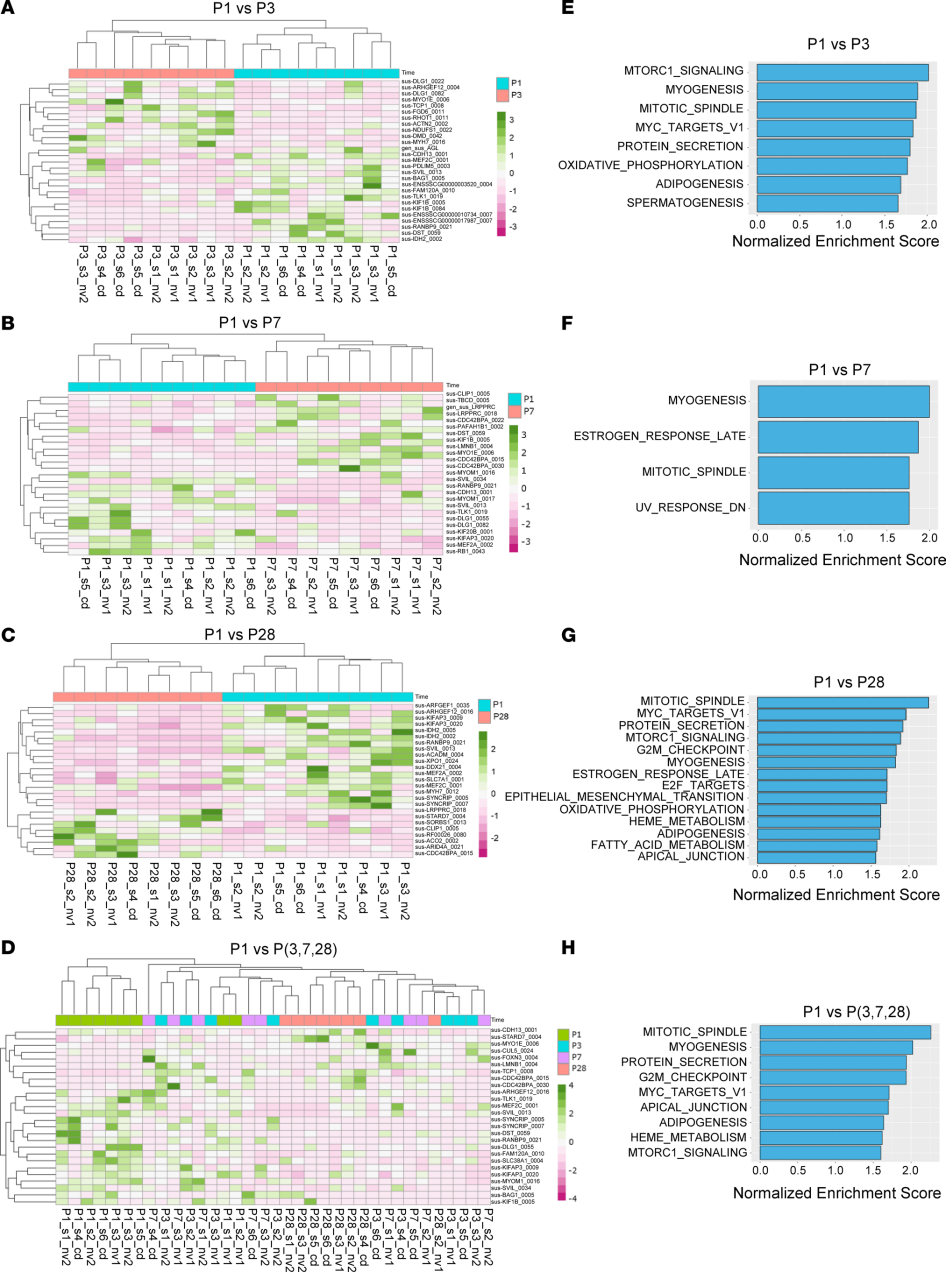
Differentially expressed circRNAs in neonatal pig hearts and functional enrichment analysis. (**A**–**D**) A heatmap of differentially expressed and cell cycle–related circRNAs depicting expression patterns from various comparisons including P1 versus P3 (**A**), P1 versus P7 (**B**), P1 versus P28 (**C**), and P1 versus P3, P7, and P28 (**D**). circRNAs with high expression values are colored in dark green while those with low expression values are in dark pink. (**E**–**H**) Pathway enrichment analysis plots of top ranked cell cycle–related hallmark pathways from various comparisons including (**E**) P1 versus P3, (**F**) P1 versus P7, (**G**) P1 versus P28, and (**H**) P1 versus P3, P7, and P28. Panels **A**–**H** were generated using circRNA data from 35 batch-corrected RNA samples obtained from 24 pig hearts sequenced by CD Genomics Inc. (“cd”), and Novogene Inc. (“nv1” and “nv2”).

Differentially expressed (i.e., upregulated or downregulated) circRNAs were identified from various comparisons (i.e., P1 versus P3, P1 versus P7, P1 versus P28, and P1 versus P3, P7, and P28; Supplemental Figure 2B), and the highest number of differentially expressed circRNAs were observed in the comparison between P1 and P28. Volcano plots were generated to provide a comprehensive view of the differential expression patterns (Supplemental Figure 2, C–F). To understand potential involvement of circRNA host genes in cell cycle regulation, circRNAs that were enriched in cell cycle–related pathways were identified and utilized to generate heatmaps. These heatmaps displayed the expression patterns of the differentially expressed and top ranked cell cycle–related circRNAs across different ages (Figure 1, A–D). Pathway enrichment analysis of the hallmark gene sets from the Human Molecular Signatures Database (40) demonstrated that differentially expressed circRNAs were enriched in different pathways associated with cell cycle activities, such as mitotic spindle assembly and myogenesis, cell cycle checkpoint, and the E2F or Myc target genes (P1 versus P3, Figure 1E; P1 versus P7, Figure 1F; P1 versus P28, Figure 1G; and P1 versus P3, P7, and P28, Figure 1H). The leading edge genes associated with these pathways were listed in Supplemental Table 2. These findings demonstrated significant changes in pathways related to cell division, mitotic spindle assembly, skeletal muscle development, and protein secretion due to differential circRNA expression. Taken together, these data provided detailed information about circRNA expression profiles in neonatal pig hearts, indicating their potential involvement in heart development.

### Time-varying effect modeling of cell cycle–regulating circRNAs.

A notable advantage of the current study is that we examined the expression profile of circRNA in pig hearts at different postnatal ages. To evaluate the overall time dependency of circRNA expression in cell cycle–related functions, we utilized our circRNA data set to analyze how the expression of different cell cycle–enriched sets of circRNA host genes varied across different postnatal days. This was achieved by time-varying effect modeling (TVEM) analysis of the host genes described above. For each of the selected cell cycle–related pathways, we then reported and visualized the changes in the β_1_ coefficient across different postnatal periods (Supplemental Figure 3). We observed significant variation in the β_1_ coefficients over time in core cell cycle–related pathways, such as G2M checkpoint (Supplemental Figure 3A), mitotic spindle (Supplemental Figure 3B), Myc-targets (Supplemental Figure 3, C and D), myogenesis (Supplemental Figure 3E), PI3K-Akt-mTOR signaling (Supplemental Figure 3F), and Wnt-beta catenin signaling (Supplemental Figure 3G), suggesting that circRNAs potentially play a crucial role in regulating cell cycle during postnatal heart development.

### Differential expression analysis of miRNAs and mRNAs in neonatal pig hearts.

**Figure 2:**
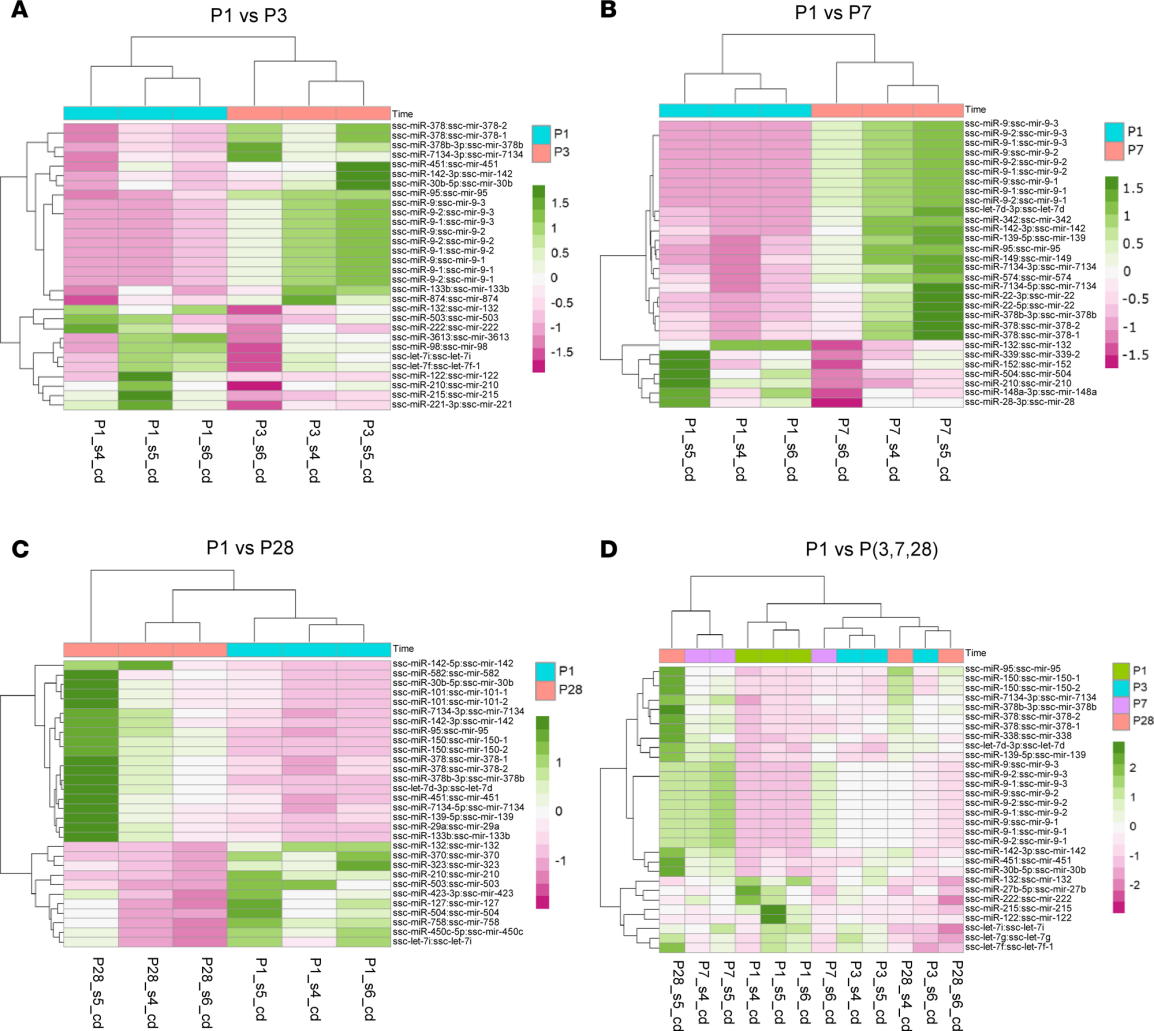
Differentially expressed miRNAs in neonatal pig hearts and functional enrichment analysis. (**A**–**D**) A heatmap of top 30 differentially expressed miRNAs depicting time-specific expression patterns in different experimental designs including (**A**) P1 versus P3, (**B**) P1 versus P7, (**C**) P1 versus P28, and (**D**) P1 versus P3, P7, and P28. circRNAs with high expression values are colored in dark green while those with low expression values are colored in dark pink. The heatmap plots displayed indicate noticeable differences in expression patterns (either higher or lower) at P1 compared with the other days, suggesting a significant alteration in the expression of miRNAs during the postnatal development of pig hearts. All panels were generated using miRNA data from 12 RNA samples obtained from 12 pig hearts at different postnatal days (P1, P3, P7, and P28).

**Figure 3:**
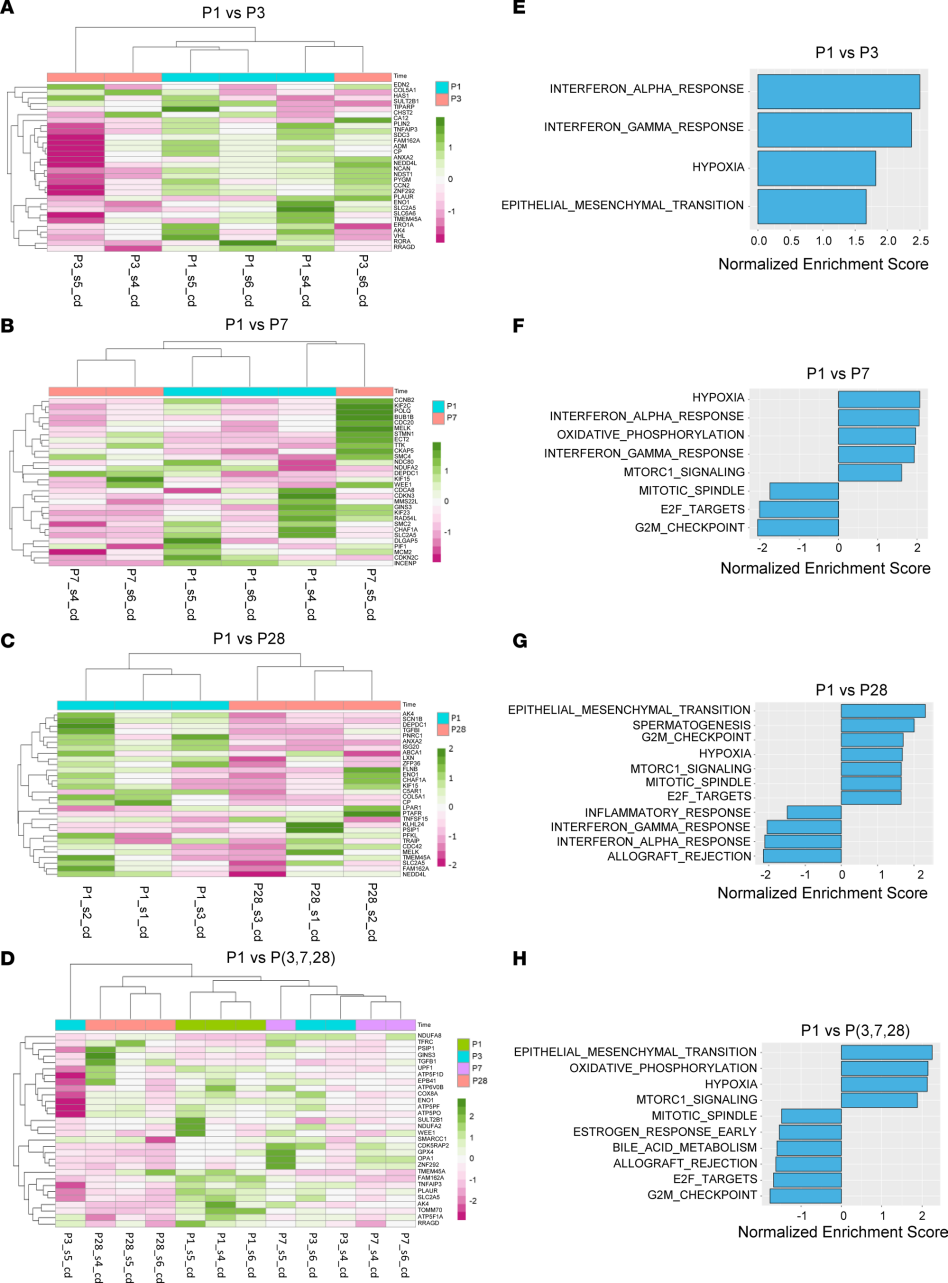
Differentially expressed mRNAs in neonatal pig hearts and functional enrichment analysis. (**A**–**D**) A heatmap of top 30 differentially expressed and cell cycle–related mRNAs depicting expression patterns from various comparisons including (**A**) P1 versus P3, (**B**) P1 versus P7, (**C**) P1 versus P28, and (**D**) P1 versus P3, P7, and P28. mRNAs with high expression values are colored in dark green while those with low expression values are in dark pink. (**E**–**H**) Pathway enrichment analysis plots of significantly enriched hallmark pathways from various comparisons including (**E**) P1 versus P3, (**F**) P1 versus P7, (**G**) P1 versus P28, and (**H**) P1 versus P3, P7, and P28. Pathways significantly enriched (FDR < 0.05) are colored in green. All panels were generated using miRNA data from 12 RNA samples obtained from 12 pig hearts at different postnatal days (P1, P3, P7, and P28).

We did not have a good batch correction for miRNA-sequencing data from batches cd and nv2. Therefore, we analyzed the expression profiles of miRNAs and mRNAs using data from batch cd only. The PCA plots of sequencing data for miRNA (Supplemental Figure 4A) and mRNA (Supplemental Figure 5A) showed clear distinctions among the 4 groups. The number of differentially expressed miRNAs in various comparisons (i.e., P1 versus P3, P1 versus P7, P1 versus P28, and P1 versus P3, P7, and P28) was provided for miRNA (Supplemental Figure 4B) and mRNA (Supplemental Figure 5B), and the highest number of differentially expressed miRNAs and mRNAs were observed in the comparison between P1 and P28. Volcano plots were generated to present a complete overview of the differential expression profiles for miRNAs (Supplemental Figure 4, C–F) and mRNAs (Supplemental Figure 5, C–F). Heatmaps were generated to display the expression patterns for the top ranked differentially expressed miRNAs (Figure 2, A–D) and mRNAs (Figure 3, A–D) associated with cell cycle. This highlighted the levels of intensity across various samples and showed the time dependency in the expression of these RNAs. The pathway analyses of mRNA data resulted in enrichment of various pathways, such as inflammation, responses to hypoxia, epithelial-mesenchymal transition, oxidative phosphorylation, mTORC1-related genes, and cell cycle–targeted genes (P1 versus P3, Figure 3E; P1 versus P7, Figure 3F; P1 versus P28, Figure 3G; and P1 versus P3, P7, and P28, Figure 3H). The resulting top leading edge associated with these pathways is listed in Supplemental Table 3. As can be inferred from the information above, a notable difference in the comparison between P1 and other groups is the presence of pathways that are associated with cell cycle and metabolism (Figure 3, E–H).

### Validation of circRNA expression in neonatal pig hearts.

**Figure 4:**
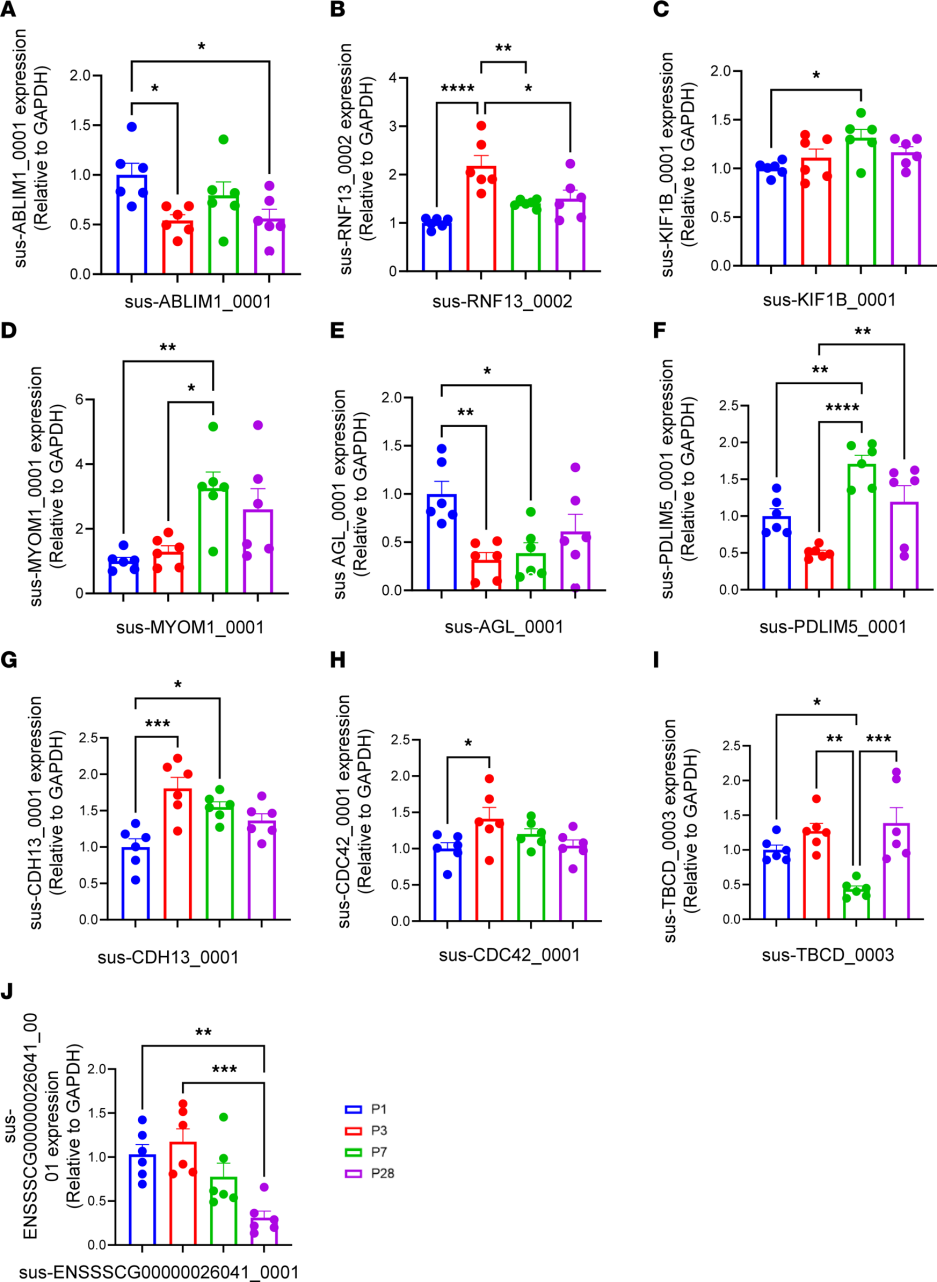
Validation of circRNA expression in neonatal pig hearts. (**A**–**J**) qRT-PCR was performed to validate the expression of 10 selected circRNAs associated with the cell cycle regulation pathway in neonatal pig hearts. All data were presented as mean ± SEM. Statistical comparisons between 2 groups in multiple groups were evaluated by 1-way ANOVA with Tukey’s honestly significant difference test for panels **A**–**J**. *n* = 6 pig hearts for each group. *P < 0.05, **P < 0.01, ***P < 0.001, and ****P < 0.0001 when comparing the expression levels between them. The relative expression levels of circRNAs were normalized to GAPDH reference gene.

We validated the expression of top ranked differentially expressed circRNAs in cell cycle–related pathways from the RNA sequencing using qRT-PCR on different ages of pig heart samples. Ten circRNAs showed consistent results between qRT-PCR and circRNA sequencing across all time points (P1, P3, P7, and P28), including sus-ABLIM1_0001, sus-RNF13_0002, sus-KIF1B_0001, sus-MYOM1_0001, sus-AGL_0001, sus-PDLIM5_0001, sus-CDH13_0001, sus-CDC42_0001, sus-TBCD_0003, and sus-ENSSSCG00000026041_0001 (Figure 4, A–J). Expression of some circRNAs were initially increased and then decreased as the pigs matured, such as sus-RNF13_0002 (Figure 4B), sus-KIF1B_0001 (Figure 4C), sus-MYOM1_0001 (Figure 4D), sus-PDLIM5_0001 (Figure 4F), sus-CDH13_0001 (Figure 4G), sus-CDC42_0001 (Figure 4H), sus-TBCD_0003 (Figure 4I), and sus-ENSSSCG00000026041_0001 (Figure 4J). However, some circRNAs did not show a consistent expression pattern aligned with cardiomyocyte maturation, such as sus-ABLIM1_0001 (Figure 4A) and sus-AGL_0001 (Figure 4E). We used the circAtlas (v2) database to match pig circRNAs to their corresponding pig chromosomes, except for sus-ENSSSCG00000026041_0001, for which we could not find a matched sequence in this database. The validated circRNA information obtained from the circAtlas (v2) database is listed in Supplemental Table 4. The results obtained from qRT-PCR, which measures expression levels, were consistent with the patterns observed in RNA sequencing. This confirms the accuracy of our findings.

### circRNAs that regulate cell cycle.

**Figure 5:**
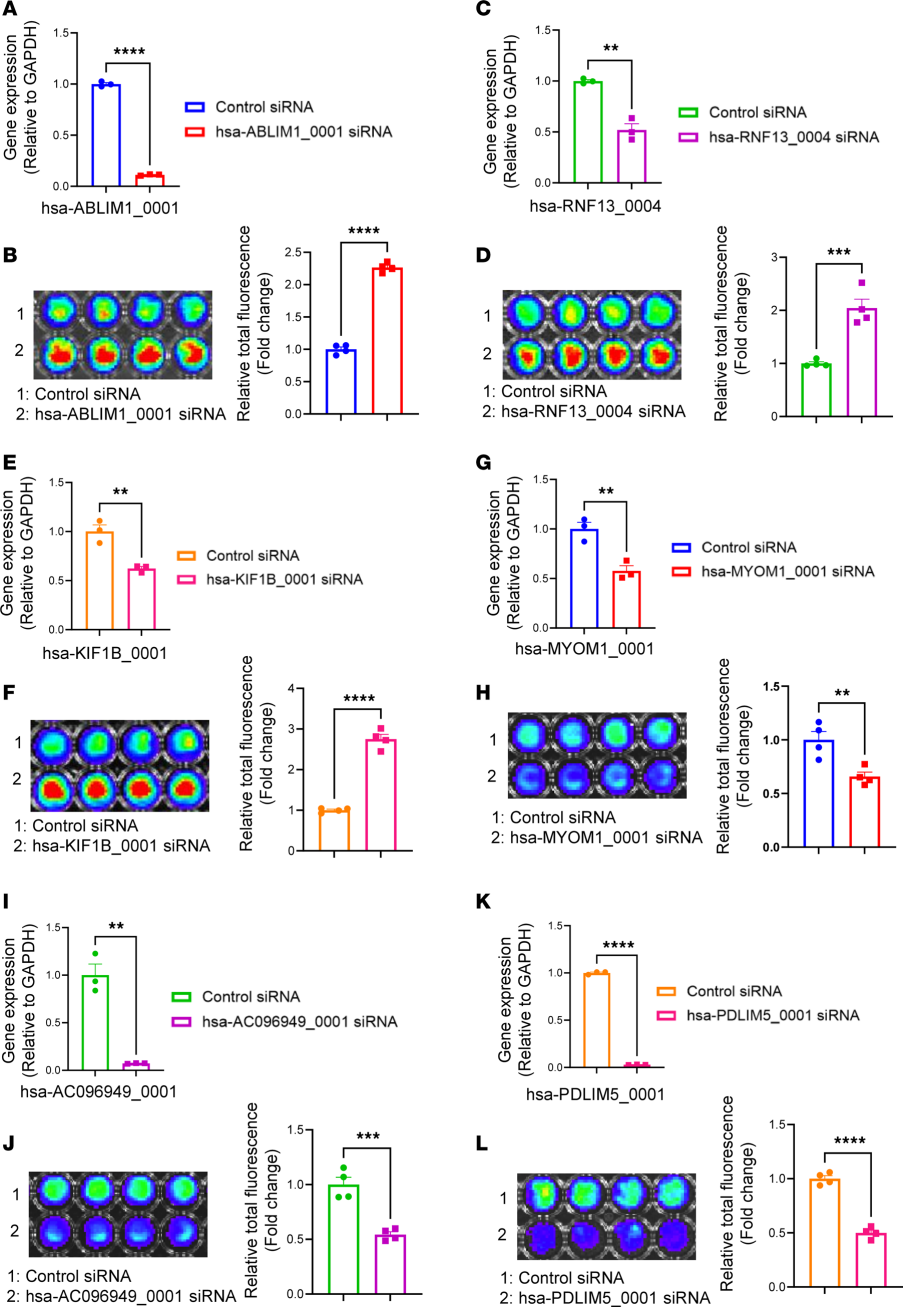
circRNAs that modulate cardiomyocyte cell cycle. hiPSC-CMs at day 28 after initiation of cardiac differentiation were utilized. The efficiency of siRNA-based knockdown of circRNAs in hiPSC-CMs was determined by qRT-PCR. The relative expression levels of circRNAs were normalized to GAPDH reference gene. Cell number was evaluated by bioluminescence analysis. Data were presented as percentage. (**A**, **C**, **E**, **G**, **I**, and **K**) Evaluation of the expression of hsa-ABLIM1_0001, hsa-RNF13_0004, hsa-KIF1B_0001, hsa-MYOM1_0001, hsa-AC096949_0001, and hsa-PDLIM5_0001 in hiPSC-CMs after siRNA treatment. (**B**, **D**, **F**, **H**, **J**, and **L**) Bioluminescence assay and quantification of bioluminescent signal intensity in hiPSC-CMs with siRNA-based knockdown of the specific circRNAs. All data were presented as mean ± SEM. Statistical analysis was performed via 2-tailed Student’s *t* test. *n* = 3 technical replicates in each group for panels **A**, **C**, **E**, **G**, **I**, and **K**. *n* = 4 technical replicates in each group for panels **B**, **D**, **F**, **H**, **J**, and **L**. **P < 0.01, ***P < 0.001, and ****P < 0.0001.

**Figure 6:**
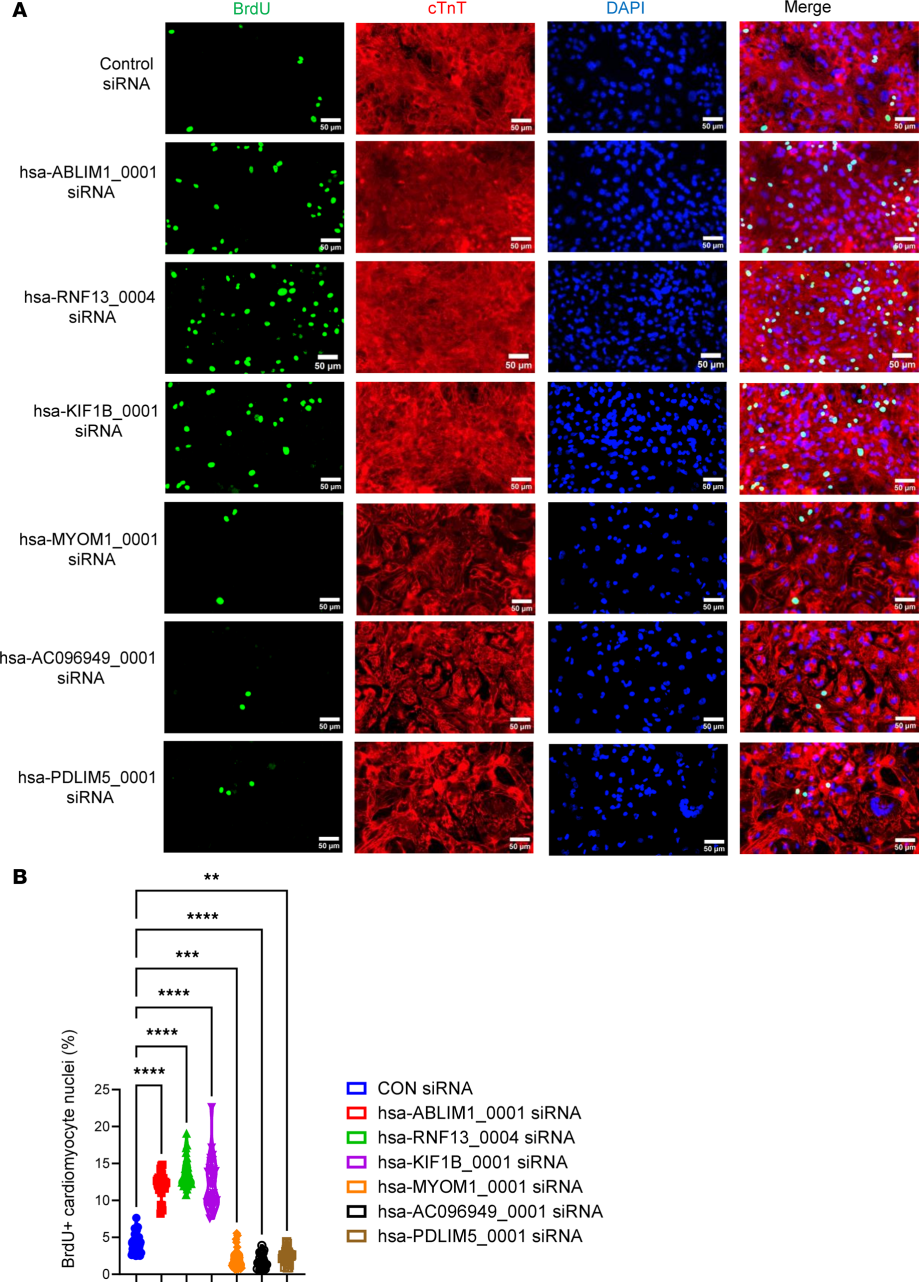
Evaluation of cardiomyocyte cell cycle via BrdU incorporation assay. hiPSC-CMs at day 28 after initiation of cardiac differentiation were used. Cell cycle was assessed by immunostaining using antibodies against BrdU (to label cells in S phase). Cardiomyocytes were labeled using anti-human cardiac troponin T (cTnT) immunostaining; all nuclei were counterstained with DAPI. The prevalence of BrdU positively stained nuclei of hiPSC-CMs was counted and normalized to the total number of cardiomyocyte nuclei. Data were presented as percentage. (**A**) Representative images of anti-BrdU immunostaining in hiPSC-CMs. (**B**) Quantification of the prevalence of BrdU positively stained nuclei of hiPSC-CMs after siRNA-based knockdown. All data were presented as mean ± SEM. Statistical analysis was performed via 1-way ANOVA with Tukey’s honestly significant difference test. *n* = 30 technical replicates in each group. **P < 0.01, ***P < 0.001, and ****P < 0.0001.

**Figure 7:**
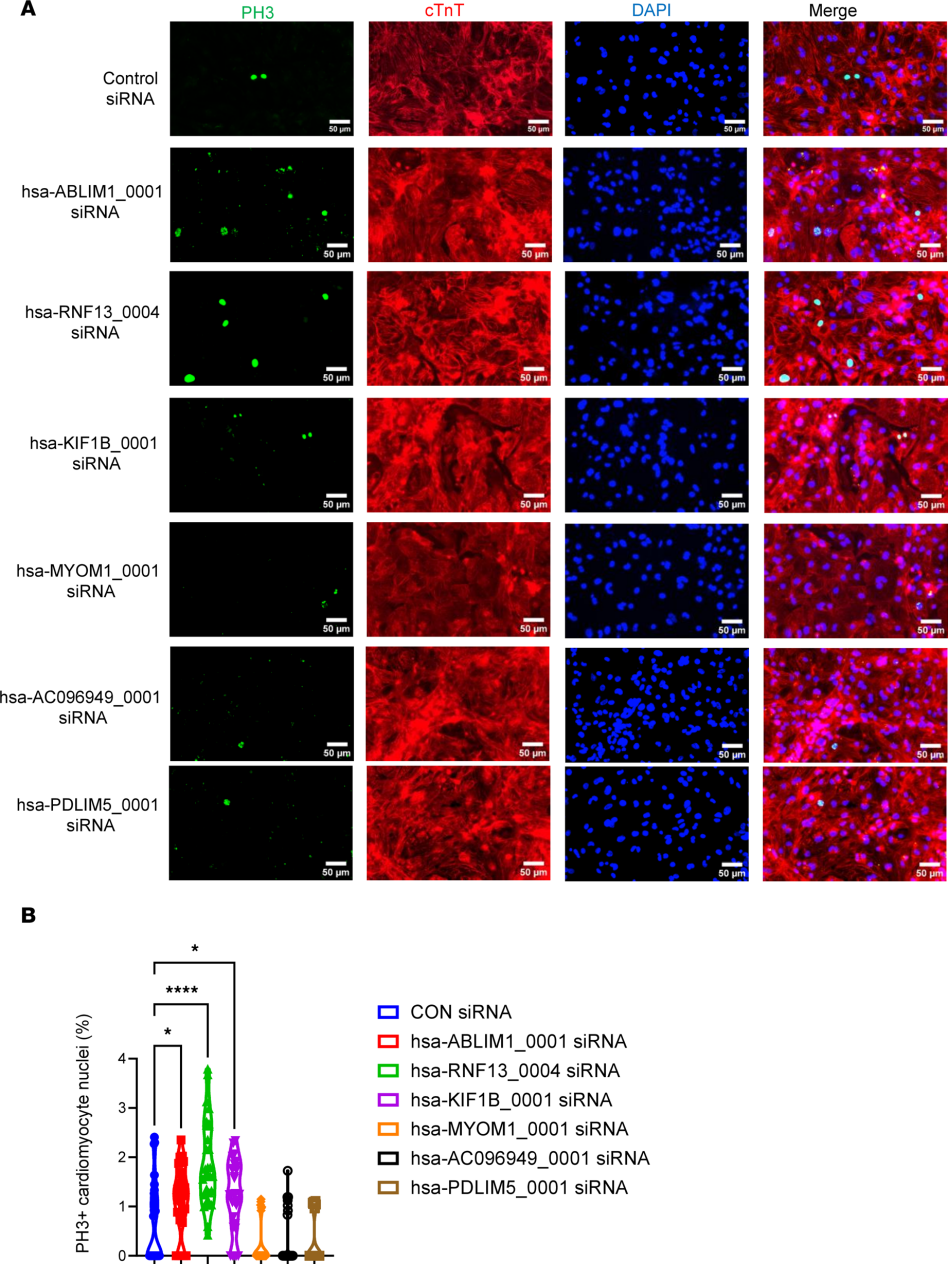
Evaluation of cardiomyocyte cell cycle via immunostaining for phosphorylated histone H3. Cell cycle was assessed by immunostaining using antibodies against phosphorylated histone H3 (PH3) (to label cells in mitosis phase). Cardiomyocytes were labeled using anti-human cTnT immunostaining; all nuclei were counterstained with DAPI. The prevalence of PH3 positively stained nuclei of hiPSC-CMs was counted and normalized to the total number of cardiomyocyte nuclei. Data were presented as percentage. (**A**) Representative images of anti-PH3 immunostaining in hiPSC-CMs. (**B**) Quantification of the prevalence of PH3 positively stained nuclei of hiPSC-CMs after siRNA-based knockdown. All data were presented as mean ± SEM. Statistical analysis was performed via 1-way ANOVA with Tukey’s honestly significant difference test. *n* = 30 technical replicates in each group. *P < 0.05, and ****P < 0.0001.

We conducted a phenotypic validation of the functions of the 9 circRNAs (Supplemental Table 4, except for sus-ENSSSCG00000026041_0001, for which we could not find a matched sequence in the database) using human induced pluripotent stem cell–derived cardiomyocytes (hiPSC-CMs), as there are no appropriate pig cardiomyocyte lines available. To accomplish this, we initially matched pig circRNAs to their respective human circRNAs using the circAtlas (v2) database. We successfully matched 6 pig circRNAs with human circRNAs, namely hsa-ABLIM1_0001, hsa-RNF13_0004, hsa-KIF1B_0001, hsa-MYOM1_0001, hsa-AC096949_0001, and hsa-PDLIM5_0001 (Supplemental Table 5). We then assessed the impact of siRNA-based knocking down of these 6 circRNAs on the cell cycle of hiPSC-CMs (Figures 5, 6, and 7). These cells carry a luciferase gene, allowing us to estimate the cell number through a bioluminescence assay as previously described (41–43). Our data showed that reducing the expression of hsa-ABLIM1_0001 (Figure 5A), hsa-RNF13_0004 (Figure 5C), and hsa-KIF1B_0001 (Figure 5E) resulted in increased cell cycle activity, as demonstrated by a 2- to 3-fold rise of fluorescence intensity in the bioluminescence assays (Figure 5, B, D, and F, respectively), along with a 3- to 4-fold rise in the proportion of hiPSC-CMs positively stained with BrdU (an S phase maker) and PH3 (an M phase marker), compared with cells in the control group (Figure 6, A and B; and Figure 7, A and B, respectively). Conversely, inhibiting the expression of hsa-MYOM1_0001 (Figure 5G), hsa-AC096949_0001 (Figure 5I), and hsa-PDLIM5_0001 (Figure 5K) resulted in decreased cell cycle activity, as shown by a 50% reduction in fluorescence intensity in the bioluminescence assays (Figure 5, H, J, and L, respectively) and a 50%–70% decrease in the proportion of BrdU and PH3 positively stained hiPSC-CMs compared with cells in the control group (Figure 6, A and B; and Figure 7, A and B, respectively). It was reported that mature miRNAs and mRNAs primarily function in the cytoplasm (44), and circRNAs regulate gene expression by binding to miRNAs (4). In our study, we examined the intracellular localization of 6 circRNAs that we have shown to regulate the cell cycle. Our data demonstrated that all these circRNAs were predominantly located in the cytoplasm, particularly in the perinuclei region, with some also present in the nuclei of hiPSC-CMs (Supplemental Figure 6, A–F).

### circRNA-miRNA-mRNA network construction.

**Figure 8:**
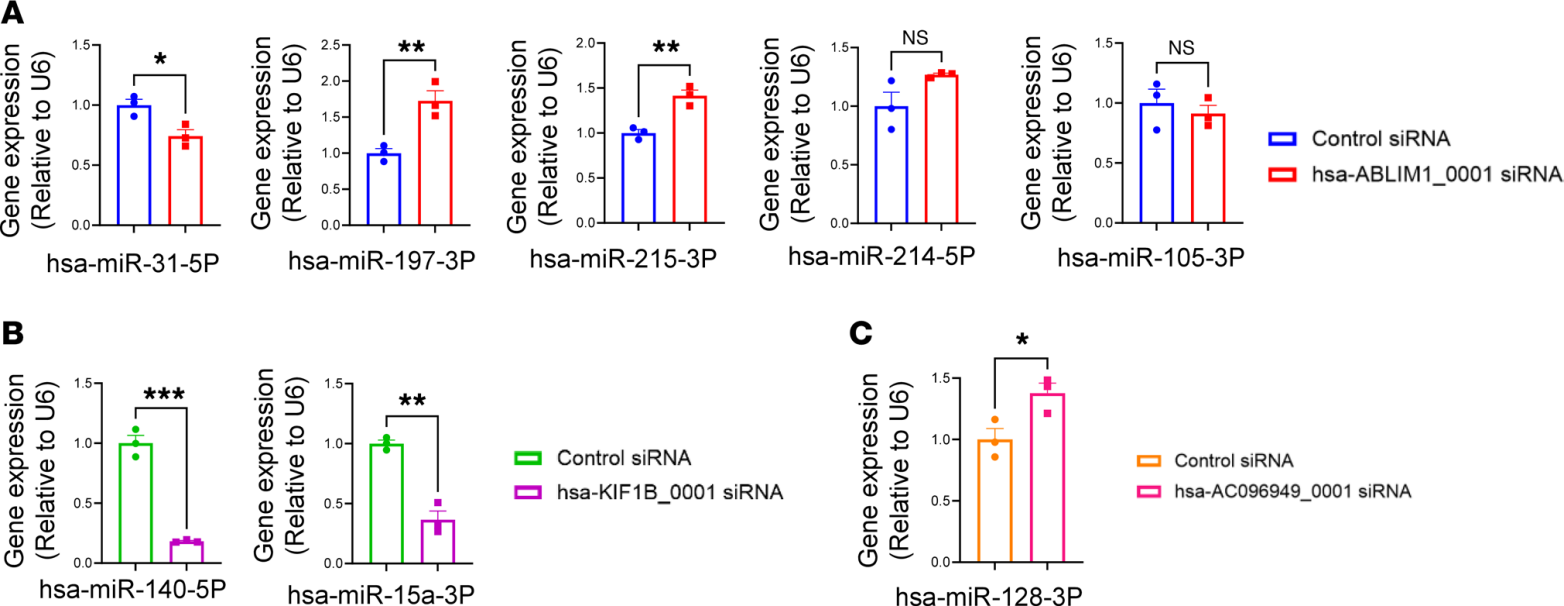
Impact of circRNA knockdown on the expression of miRNAs. hiPSC-CMs at day 28 after the initiation of cardiac differentiation were used. qRT-PCR was used to evaluate the expression level of various miRNAs in hiPSC-CMs. This included the evaluation of hsa-miR-31-5P, hsa-miR-197-3P, hsa-miR-215-3P, hsa-miR-214-5P, and hsa-miR-105-3P in hiPSC-CMs treated with hsa-ABLIM1_0001 siRNA (**A**), hsa-miR-140-5P and hsa-miR-15a-3P in hiPSC-CMs treated with hsa-KIF1B_0001 siRNA (**B**), and hsa-miR-128-3P in hiPSC-CMs treated with hsa-AC096949_0001 siRNA (**C**). The relative expression levels of miRNAs were normalized to U6 reference gene. All data were presented as mean ± SEM. Statistical analysis was performed via Student’s 2-tailed *t* test. *n* = 3 technical replicates in each group. *P < 0.05, **P < 0.01, and ***P < 0.001.

To better understand the potential molecular mechanism of circRNAs involved in the process of cell proliferation, circRNAs and their predicted targets at different stages of heart development were used to construct a circRNA-miRNA-mRNA regulatory network (Supplemental Figure 7, A–C). Specifically, we selected 5 pig circRNAs that were conserved in humans, including sus-ABLIM1_0001, sus-RNF13_0002, sus-KIF1B_0001, sus-AGL_0001, and CDH13_0001. Using circAtlas (v2), we predicted a total of 11 target miRNAs. A total of 45 target mRNAs for the 11 miRNAs were then predicted using miRNAtap (45) package in R. To infer potential circRNA-miRNA-mRNA relationships, we used Cytoscape software to generate circRNA-miRNA-mRNA networks (46). These RNA interactions provided new insights into the potential mechanism for cell proliferation. Specifically, we predicted interactions such as sus-ABLIM1_0001 with miR-31-5p, miR-105-3p, miR-197-3p, miR-214-5p, miR-215-3p (Supplemental Figure 7, A–C), and miR-105-3P (Supplemental Figure 7C); sus-KIF1B_0001 with miR-140-5p, miR-18a-3p, and miR-15a-3p (Supplemental Figure 7, A–C); sus-RNF13_0002 with miR-7-5p (Supplemental Figure 7, A–C); sus-CDH13_0001 with miR-132-5p (Supplemental Figure 7, A–C); and sus-AGL_0001 with miR-128-3p (Supplemental Figure 7C). To validate these predictions and determine if circRNAs regulate the expression of these miRNAs, we treated hiPSC-CMs with siRNAs targeting the circRNAs. We then examined the impact of circRNA knockdown on the expression levels of the predicted miRNAs. Our data showed that, compared with hiPSC-CMs treated with a control siRNA, those treated with hsa-ABLIM1_0001 siRNA showed a significant increase of miR-31-5p, miR-197-3p, and miR-215-3p, while the expression levels of miR-214-5p and miR-105-3p remained unchanged (Figure 8A). Additionally, hiPSC-CMs treated with hsa-KIF1B_0001 siRNA exhibited a significant decrease of miR-140-5p and miR-15a-3p compared with the control (Figure 8B). On the other hand, hiPSC-CMs treated with hsa-AC096949_0001 (the human counterpart of pig circRNA sus-AGL_0001) siRNA displayed a significant increase of miR-128-3p compared with the control (Figure 8C). Unfortunately, we were not able to detect miR-7-5p after the transfection of siRNAs targeting the circRNA RNF13_0002. In addition, the siRNAs for circRNA hsa-CDH13_0001 did not work for unknown reasons (data not shown).

To determine if modulating the expression of these miRNAs alters cardiomyocyte cell cycle, hiPSC-CMs were treated with miRNA siRNAs or mimics, respectively. Bioluminescence assays were used to estimate the number of hiPSC-CMs. Our data indicated that inhibiting miR-31-5p, miR-197-3p, or miR-215-3p using different concentrations of miRNA siRNAs (0, 1, 5, 10, 20, and 100 nM) resulted in reduced cell cycle of hiPSC-CMs in a dose-dependent manner (Supplemental Figure 8, A–C). Additionally, a low dose (1 nM) of miR-197-3p mimics promoted cell cycle, whereas high doses of miR-197-3p and miR215-3p mimics (100 nM) reduced cell cycle. Furthermore, inhibiting miR-140-5p with a high dose of miRNA siRNAs or miR-15a-3p with various concentrations of miRNA siRNAs resulted in a reduced cell cycle of hiPSC-CMs. Similarly, a high dose of miR-140-5p siRNAs and various concentrations of miR-140-5p mimics also led to a reduction of the cell cycle (Supplemental Figure 8D). Moreover, when low doses (1 and 5 nM) of miRNA siRNAs were used to inhibit miR-128-3p, it promoted the cell cycle of hiPSC-CMs (Supplemental Figure 8F). However, high doses of miRNA siRNAs and activation with different concentrations of mimics both led to a decreased cell cycle of hiPSC-CMs.

Expression of miR-128 increases in postnatal mouse hearts. Overexpression of miR-128 inhibits and knockout of miR-128 promotes the cell cycle of neonatal mouse cardiomyocytes (47). The function of miR-128 in pig or human cardiomyocytes remains unknown. Therefore, we determined the impact of inhibition or activation of miR-128-3p on the expression of “upstream” circRNA and “downstream” mRNAs using qRT-PCR and on the cell cycle of hiPSC-CMs via bioluminescence assay and immunostaining using antibodies against BrdU and PH3. qRT-PCR verified the increased expression of miR-128-3p in hiPSC-CMs treated with 5 nM of hsa-miR-128-3p mimics, which resulted in reduced expression of hsa-AC096949_0001 (Supplemental Figure 9, A and B). Additionally, hsa-miR-128-3p mimics decreased the cell cycle of hiPSC-CMs, as indicated by the reduced bioluminescent signal compared with control, along with a decrease in the prevalence of BrdU- and PH3-positive cells (Supplemental Figure 9, C–E). These data are consistent with our previous finding that siRNA-mediated inhibition of hsa-AC096949_0001 resulted in a decreased cell cycle of hiPSC-CMs (Figure 5, I and J; Figure 6, A and B; and Figure 7, A and B). Furthermore, hsa-miR-128-3p mimics reduced the expression of membrane metalloendopeptidase (MME) (Supplemental Figure 9F). On the other hand, inhibiting the expression of miR-128-3p in hiPSC-CMs treated with 5 nM of hsa-miR-128-3p siRNAs resulted in increased expression of hsa-AC096949_0001 (Supplemental Figure 10, A and B). Additionally, hsa-miR-128-3p siRNAs increased the cell cycle of hiPSC-CMs as shown by the increased bioluminescent signal compared with control, as well as an enhanced prevalence of BrdU- and PH3-positive cells, which suggested an increased cell cycle (Supplemental Figure 10, C–E). Furthermore, hsa-miR-128-3p mimics increased the expression of MME (Supplemental Figure 10F). In the future, more research is needed to study the cell cycle function of MME and to validate other miRNAs and mRNAs in the circRNA-miRNA-mRNA network to identify new pathways for regulating cardiomyocyte proliferation and regeneration. Overall, these results suggest the potential effects of RNA interactions on the regulation of cardiomyocyte cell cycle.

## Discussion

In recent years, there has been an increasing amount of evidence indicating the important role of circRNAs in various physiological and pathological processes related to human health and disease (48–50). However, the expression and function of circRNAs in the hearts of large mammals are largely unknown. In the current study, we examined the expression patterns of circRNAs in pig hearts at different postnatal stages (P1, P3, P7, and P28) (Figure 1). circRNAs that were found to be differentially expressed in this study were mainly linked to host genes that are involved in a variety of processes, including cell division, apoptosis, angiogenesis, immune responses, muscle development, transition between epithelial and mesenchymal cells, and metabolism (Figure 1, E–H). Some of these pathways are closely linked to cardiomyocyte cell cycle and proliferation, which are pivotal for myocardial regeneration.

Cardiomyocyte cell cycle and heart regeneration are intricate processes that involve the coordinated expression of numerous genes and regulatory molecules. Recently, circRNAs have emerged as important contributors in these processes, functioning as essential regulators of gene expression through various mechanisms. Huang et al. investigated the roles of circNfix in adult mice after myocardial infarction (10). Their data suggested that inhibition of circNfix promoted cardiomyocyte proliferation and improved heart function. Si et al. discovered that circHipk3 overexpression promotes cardiomyocyte proliferation by increasing Notch1 intracellular domain (N1ICD) acetylation, thereby increasing the N1ICD stability and preventing its degradation. Furthermore, adeno-associated virus 9–mediated (AAV9-mediated) overexpression of circHipk3 induces cardiac regeneration and improves heart function in adult mice after myocardial infarction (11). Zhang et al. reported that circCDYL overexpression promotes proliferation of cardiomyocytes in vitro, and AAV9-mediated overexpression of circCDYL improves heart function in adult mice after myocardial infarction (12). Ma et al. reported that inhibiting the expression of circMdc1 increases cardiomyocyte cell cycle activity. In addition, the cardiac AAV-mediated knockdown of circMdc1 promotes cardiac regeneration in both neonatal and adult mice after myocardial infarction (13). These studies collectively highlight the important role of circRNAs in regulating the cell cycle of cardiomyocytes and the process of heart regeneration. By affecting gene expression through interactions with miRNAs and other regulatory molecules, circRNAs present as potential targets for therapeutic interventions aimed at improving cardiac repair and regeneration.

Our study identified 10 new circRNAs associated with the cell cycle in pig hearts, whose expression patterns changed dynamically during cardiomyocyte maturation (Figure 4). To investigate these 10 circRNAs’ roles in cardiomyocyte cell cycle regulation, we matched them with their human counterparts (Supplemental Table 5) and conducted phenotypic validation on hiPSC-CMs in culture. Six circRNAs potentially involved in cell cycle regulation were identified. Knocking down some of these circRNAs changed cell cycle activity in hiPSC-CMs (Figures 5–7). These results highlight the complex regulatory roles of circRNAs in heart development and their potential implications in myocardial regeneration. In terms of potential therapeutic strategies for cardiovascular diseases, inhibition of hsa-ABLIM1_0001, hsa-RNF13_0004, and hsa-KIF1B_0001, or overexpression of hsa-MYOM1_0001, hsa-AC096949_0001, and hsa-PDLIM5_0001, may be promising in promoting cardiomyocyte cell cycle and heart regeneration. These findings demonstrate significant changes in circRNA expression in neonatal pig hearts with remarkable regenerative abilities, highlighting the vital role of circRNAs during early postnatal heart development of pig hearts. Further investigations are needed to uncover the specific functions and mechanisms of these circRNAs in regulating cardiomyocyte cell cycle in vivo.

Mechanisms underlying the function of circRNAs in cardiomyocyte cell cycle remain unclear. It has been reported that endogenous RNAs may function by sequestering miRNAs and modulating target mRNA expression (4). In order to obtain a deeper understanding of the regulatory function of circRNAs in cell proliferation, we conducted miRNA sequencing (Figure 2) and mRNA sequencing (Figure 3) for neonatal pig hearts and constructed a comprehensive circRNA-miRNA-mRNA network using Cytoscape (46). This approach allows us to uncover the potential regulatory pathways that target the cell cycle pathways of cardiomyocytes. We predicted 11 corresponding miRNAs and 45 target mRNAs that are associated with the cell cycle function of the 5 circRNAs (Supplemental Figure 7, A–C). Specifically, interactions between circRNAs, miRNAs, and mRNAs were predicted and used to construct a circRNA-miRNA-mRNA regulatory network. Five conserved pig circRNAs (CDH13_0001, sus-ABLIM1_0001, sus-RNF13_0002, sus-KIF1B_0001, and sus-AGL_0001) and their corresponding miRNAs were analyzed using circAtlas (v2) and miRNAtap package. Cytoscape software was utilized to construct the corresponding circRNA-miRNA-mRNA regulatory network, providing insight into potential cell proliferation mechanisms. These findings suggest that these circRNAs may regulate the cell cycle through specific miRNA interactions. However, it is crucial to validate these predictions through phenotype experiments.

circRNA sequencing has been performed in heart samples from mice, rats, pigs, and humans, as well as the cardiomyocytes derived from hiPSCs or human embryonic stem cells (51–58). Liang et al. conducted total RNA sequencing in different organs (including the heart, liver, spleen, lung, kidney, ovarium, testis, skeletal muscle, and fat) of 3 adult pigs (240 days after birth) and identified 205 unique circRNAs in the heart (57). Mester-Tonczar et al. performed RNA sequencing of rRNA-depleted RNA samples from infarcted and healthy myocardium tissue samples of 6 female adult pigs (6 months old) and discovered that the expression of Circ-RCAN2 and circ-C12orf29 was significantly downregulated in infarcted tissue compared with healthy pig heart (59). However, the majority of the RNA sequencing was done on heart samples from animals or human hearts with heart failure. Thus far, there has been no RNA sequencing performed in postnatal hearts at different ages. There are several advantages in our study. First, to increase the coverage and the quality of our circRNA-sequencing data, we sent samples to 2 companies (i.e., Novogene and CD Genomics). Prior to downstream analysis, batch correction of these 2 data sets was done as described in DESeq2 Bioconductor package. Second, we conducted qRT-PCR experiments to validate RNA-sequencing data, and the results concurred with the circRNA-sequencing data (Figure 4). Existing approaches for circRNA identification primarily rely on detecting reads spanning backsplice junctions, without differentiating between linear and circular reads that align to the internal regions of circRNAs (60). In contrast, our method involves transcribed cDNA molecules containing multiple copies of the corresponding full-length circRNA sequence, and each long read provides direct evidence of a circRNA’s presence and sequence (61). Additionally, we employed qRT-PCR to validate the reliability of circRNA quantification, randomly selecting circRNAs to enhance the applicability of this approach to circRNA studies (62). To improve the accuracy of circRNA identification, we excluded candidates lacking canonical AG/GT splice sites and candidates mapped to the mitochondrial genome (63).

There are limitations in our study. First, although we have included 6 samples in each group, it would be beneficial to further increase the sample size. Second, because of the lack of a pig cardiomyocyte cell line and limited availability of pig databases for analyzing mRNA and miRNAs, we chose to use conserved human circRNAs to construct the circRNA-miRNA-mRNA network. Third, the RNA sequencing was performed using samples from the entire heart, which contain both myocytes and nonmyocytes.

In summary, data from the current study improved our understanding of the essential roles played by circRNAs in the development of pig hearts and uncovered new insights into potential mechanisms that drive cell proliferation through the exploration of circRNAs. To our knowledge, this is the first report about circRNA profiles in the postnatal hearts of large mammals. Although we acknowledge limitations such as sample variation and the need for further validation, our findings will greatly enhance our understanding of circRNA function and its potential implications in heart development and maturation in large mammals. It was reported that modulating the expression of circRNAs confers cardioprotection in preclinical animal models of heart failure (19, 64, 65). In this study, we identified and validated 6 circRNAs that regulate cardiomyocyte cell cycle and proliferation. We also matched these pig circRNAs to human circRNAs and constructed the circRNA-miRNA-mRNA network. As a next step, we will validate the potential roles of these circRNAs in regulating myocardial regeneration in pig hearts and explore potential small molecules that may regulate the expression of the circRNAs. Therefore, the successful identification of the circRNAs that control heart regeneration in pigs will provide new therapeutic targets for the treatment of heart failure in humans.

## Methods

### Sex as a biological variable.

A total of 6 animal hearts including both males and females were used from each age group, and similar findings are reported for both sexes.

### Experimental animals.

The hearts of domestic Yorkshire pigs (Premier Biosource) at P1, P3, P7, and P28 were isolated. These hearts were rinsed in cold saline immediately, then frozen in liquid nitrogen. RNA sequencing of mRNAs and noncoding RNAs including circRNAs and miRNAs was performed using these samples. qRT-PCR was then used for data validation. Information on key reagents is listed in Supplemental Table 6, and primary and secondary antibodies are in Supplemental Table 7.

### Total RNA extraction and qRT-PCR.

The RNeasy Mini Kit (QIAGEN) was used to extract total RNA from pig heart tissue and hiPSC-CMs. The concentration of RNA in the samples was determined by measuring the absorbance at 260 nm using UV spectrophotometry, specifically with the NanoDrop 2000 instrument (Thermo Fisher Scientific). The High-Capacity cDNA Reverse Transcription Kit (Applied Biosystems) was used to generate cDNA. qRT-PCR was performed using the SYBR Green PCR Master Mix (Roche). The primer sequences used in this experiment are listed in Supplemental Table 8. Each qRT-PCR was conducted in triplicate using a LightCycler 480 System instrument (Roche). To ensure accurate normalization, gene expression values were normalized to the average expression of the housekeeping gene GAPDH.

### circRNA sequencing.

The quality of the RNA samples was evaluated using High Sensitivity RNA TapeStation system (Agilent Technologies). Quantification of RNA was performed using Qubit 2.0 RNA High Sensitivity (HS) assay (Thermo Fisher Scientific). Depletion of rRNA was performed using RiboZero Gold kit based on the instructions. Next, RNaseR buffer and RNaseR were added to the rRNA-depleted samples to remove linear RNA. Construction of the library was performed according to the instructions provided by the manufacturer for the New England Biolabs NEBNext Ultra II RNA (Directional) kit. The quantity of the final library was measured using KAPA SYBR FAST qPCR, and the quality of the library was assessed using the TapeStation D1000 ScreenTape (Agilent Technologies). Illumina 8 nt dual indices were used for library indexing. Equimolar pooling of the libraries was performed based on quality control values. The libraries were subsequently sequenced on an Illumina sequencer (Illumina) with a read length configuration of 150 paired-end reads. The goal was to obtain 20 million paired-end reads per sample (20 million in each direction).

### Small RNA sequencing.

RNA sample quality was assessed using the High Sensitivity RNA TapeStation and quantified with the Qubit 2.0 RNA HS assay. Construction of the library was performed according to the instructions provided by the manufacturer for the NEBNext Small RNA Library Prep kit. In brief, the 3′ SR adapter was ligated to the RNA. Subsequently, reverse transcriptase with SR RT Primer were used to generate first-strand cDNA, followed by the ligation of the 5′ SR Adaptor and synthesis of the second-strand cDNA. The resulting products were purified and enriched through PCR to create the final cDNA library. Fragments within the range of 140–160 bp (including miRNAs with lengths of 18–40 bp and adapters/barcodes with length of 120 bp) were selected. The quantity of the final library was measured using KAPA SYBR FAST qPCR, and the library quality was assessed using the TapeStation D1000 ScreenTape. Equimolar pooling of libraries was performed based on quality control values, and sequencing was carried out on an Illumina sequencer with a read length configuration of 150 paired ends, generating 10 million paired-end reads per sample (20 million in each direction).

### Total RNA sequencing.

The quality of the RNA samples was evaluated using the High Sensitivity RNA TapeStation system. Quantification of RNA was measured utilizing the Qubit 2.0 RNA HS assay. rRNA was removed using the RiboZero Gold kit based on the manufacturer’s instructions. Library construction was performed following the manufacturer’s instructions for the TruSeq Stranded Total RNA kit. This process requires breaking down the RNA into smaller pieces by using divalent cations at higher temperatures (typically around 70°C–95°C). Cleaved RNA fragments were then reverse-transcribed into first-strand cDNA using reverse transcriptase and random primers, followed by the synthesis of second-strand cDNA. Resulting cDNA fragments were modified with a single A base and ligated with an adapter. The resulting products underwent purification and PCR enrichment to generate the final cDNA library. The size of library inserts typically ranges 120–200 bp, with a median size of 150 bp. The final library quantity was determined using KAPA SYBR FAST qPCR, and the library quality was evaluated using the TapeStation D1000 ScreenTape. Equimolar pooling of libraries was performed based on quality control values. Pooled libraries were sequenced on an Illumina sequencer with a read length configuration of 150 paired-end reads. The goal was to obtain 100 million paired-end reads per sample (20 million in each direction). Since RNA was fragmented at the beginning without size selection, it was anticipated that the subsequent library would contain both coding and noncoding RNA fragments, including long noncoding RNAs (lncRNAs). Utilization of rRNA-depleted RNAs as input enhanced the chances of maintaining noncoding RNAs in the final library. As a result, the sequencing data exhibited the presence of noncoding RNAs, enabling the analysis of both coding and noncoding transcripts, including lncRNAs.

### Identification and quantification of circRNAs.

Cross-sample quality control check using metrics like percentage of uniquely mapped reads and GC content, along with their visualization, was done using MultiQC (v1.11) (66). Index generation and paired-end alignment of each sample were performed using STAR (v2.7.9a), with the *chimSegmentationMin* parameter set to 10 (67). Ensembl’s reference genome Sscrofa10.2 was used for alignment. This process resulted in the generation of chimeric junctions for each sample. CIRCexploler2 (v2.3.5) was utilized to generate back-splice junctions through its *parse* command (68). The resulting.bed files from CIRCexploler2’s *parse* step were then used to annotate circRNAs, resulting in an output of known annotated circRNAs. To enable generation of a cross-sample comparable circRNA count matrix for downstream analysis, normalization of the number of back-spliced reads per million mapped reads of circRNA was performed. This led to the generation of 2 sets of circRNA count data (one from CD Genomics data and one from Novogene data). The majority (~97%) of our quantified circRNAs overlapped with circRNAs that have been reported before and were labeled as per the pig annotation in circAtlas (v2). The rest of the unlabeled circRNAs were given novel names. Specifically, novel circRNAs were named with first prefix annotating the species, followed by the host gene name, and last the unique ID annotated by our lab followed by a unique index. For example, sus-AGL_VLJW2 was a novel circRNA identified in our study, with first prefix indicating that it is from the pig species, located in *AGL* host gene, and the last prefix VLJW2 interpreting it as the second novel circRNA found in this specific host gene (*AGL*). In addition, we obtained quantified mRNA data with approximately 6,640 protein coding genes, and quantified miRNA expression data with approximately 338 miRNAs described in experimental design section above, for our integrated downstream analysis steps below.

### Differential expression analysis and pathway enrichment analysis.

circRNA expression data with a total of 24 pig samples from CD Genomics and Novogene were combined using bedtool’s *intersection* command (69). First, batch correction of the pig samples from Novogene and CD Genomics was carried out using DESeq2 package in R (70). The batch-corrected samples were then visualized using DESeq2’s multidimensional scaling (MDS) plot and Linnorm’s hierarchical clustering of samples plot (71). Based on the MDS plot, 1 sample from Novogene on day 28 failed to correct for the batch effect and was excluded. Lowly expressed circRNAs were filtered, retaining only circRNAs with at least 10% of the samples with counts of at least 3. This resulted in approximately 7,000 filtered and batch-corrected circRNAs. To investigate differences in circRNA expression across neonatal pigs’ developmental stages, we conducted differential expression analysis of circRNA host genes under different experimental designs: day 1 versus day 3 (P1 versus P3), day 1 versus day 7 (P1 versus P7), day 1 versus day 28 (P1 versus P28), and day 1 versus days 3, 7, and 28 (P1 versus P3, P7, and P28). This differential analysis was performed using R packages DESeq2 and Linnorm. Volcano plots were generated using ggplot2 package in R for each defined experimental design above (72). circRNAs with a log 2-fold change were considered upregulated if log_2_FC > 0.5, or downregulated if log_2_FC < –0.5. If a circRNA was upregulated or downregulated and had a *P* ≤ 0.05, it was considered significant. Since both mRNA and miRNA data were sequenced in 1 batch and from 1 company (CD Genomics), batch correction was skipped. Last, differential expression analysis was repeated for our miRNA data and mRNA data, using the same experimental designs as described for circRNA above. Results from differential expression analysis of our circRNA and mRNA data were used for pathway enrichment analysis. Specifically, to investigate enriched pathways during different stages of heart development and to identify the corresponding transcripts associated with these enrichments, pathway enrichment analysis of circRNA host genes and of mRNAs was conducted using the R packages ReactomePA and ClusterProfiler (73, 74).

### Prediction of circRNA to the targeted miRNAs and miRNAs.

The majority (~97%) of our quantified circRNAs were annotated as per circAtlas (v2). This database not only provides the circRNA of interest but also includes all other reported conserved circRNAs from 6 species, including pig, mouse, and human. Additionally, the database provides information about the tissue in which the circRNA of interest is expressed. Because resources for predicting/annotation of pig circRNA to their miRNA target(s) are currently unavailable, for our selected circRNAs of interest, we utilized corresponding conserved human circRNA as annotated in circAtlas (v2). As such, for the pig circRNAs conserved in human, circAtlas (v2) was used to link them to their corresponding miRNAs detected in miRanda (75) and TargetScan (76).

Using the predicted miRNA targets above, corresponding target gene(s) were predicted using the miRNAtap (v1.6.0) package in R (45). Specifically, the function *getPredictedTargets(min_src=2)* in miRNAtap was used to predict target genes using 5 aggregation databases: miRanda (75), TargetScan (76), DIANA (77), PicTar (78), and miRDB (79), with the parameter *min_src=2* ensuring high-confidence prediction of targets reported in at least 2 of these databases. The top 54 high scoring mRNA targets were selected. Finally, of these predicted miRNAs to mRNA target genes, a filtering was done to retain those expressed in our mRNA data.

### Culture and differentiation of hiPSCs.

The hiPSC line was purchased from WiCells (catalog FTDL-01, WI), then genetically modified to express a luciferin reporter gene under a cardiac troponin T promoter as we described previously (80). These hiPSCs were cultured on Matrigel Membrane Matrix (Thermo Fisher Scientific, catalog CB356253) using mTeSR Plus basal medium supplemented with appropriate supplements until 80%–100% confluent. Subsequently, the hiPSCs were differentiated into CMs using the protocol we described before (80). In brief, the cells were cultured in basal medium for 24 hours, which consisted of RPMI 1640 medium supplemented with 2% B27 supplement minus insulin, along with CHIR99021 (a GSK-3 inhibitor). Then, 2 mL and 1 mL more basal medium were added separately in the next 2 days. After 72 hours of culture, IWR-1 (a Wnt inhibitor) was added, and the cells were cultured for an additional 48 hours. The medium was then changed every 2 days. On day 9, the medium was replaced with RPMI 1640 medium containing 2% B27 supplement. hiPSC-CMs typically began to beat at 9–12 days after the initiation of differentiation. The hiPSC-CMs were purified using metabolic selection as we described before (80). In brief, the medium was replaced with glucose-free RPMI 1640 medium supplemented with 2% B27, and the medium was changed every 3 days until 4 weeks after the initiation of cardiac differentiation. This method produced hiPSC-CMs with a purity exceeding 90% as we reported before (80).

### siRNA-based knocking down of circRNAs.

Four weeks after differentiation, hiPSC-CMs were plated either in 96-well plates (for bioluminescence analysis, 50,000 cells per well) or 2-well chamber slides (for histology studies, 500,000 cells per well), respectively. The cells were incubated in medium (RPMI 1640 medium containing 2% B27 supplement) at 37°C for 48 hours. The transfection mixture was prepared by supplementing the fresh culture medium with the transfection reagents (Lipofectamine RNAiMAX) and either negative control siRNA or siRNAs targeting specific circRNAs. The concentrations of siRNAs used were 10, 40, or 80 nM (as shown in Supplemental Table 9). On the day of siRNA transfection, the cells were washed once with PBS, then incubated with transfection mixture. Both the transfection reagent and siRNA at different concentrations were diluted 1:1 in serum-free Opti-MEM and incubated for 5 minutes at room temperature before being used to treat the cells. Functional experiments (bioluminescence analysis and histology studies) were conducted 3 days after siRNA treatments. Efficiency of siRNA-based circRNA knockdown was evaluated using qRT-PCR using primers listed in Supplemental Table 8. The siRNA experiments in Figures 6 and 7 shared the same controls.

### Evaluation of cardiomyocyte cell cycle via bioluminescence assay and immunohistology.

After the miRNA siRNA or mimic transfection, bioluminescence assays were performed by adding the d-Luciferin reagents and measuring bioluminescence signal intensity using the IVIS-100 In Vivo Imaging System (PerkinElmer) as we described before (41, 42, 80). The radiance signals obtained from the bioluminescence assay were recorded for each group of cells. Next, a standard curve was generated by plotting a linear regression curve of cell numbers versus the respective radiance obtained from the bioluminescence assay for cell densities. The standard curve allowed us to determine the cell number from the bioluminescence signal obtained from each well of the 96-well plate.

For immunohistology, the cells were fixed in 4% paraformaldehyde for 15 minutes, followed by permeabilization with 0.25% Triton X-100 for another 15 minutes at room temperature. The samples were incubated with blocking solution (10% donkey serum in Dulbecco’s PBS, pH 7.4) for 1 hour at room temperature. Subsequently, the samples were incubated with primary antibodies (as shown in Supplemental Table 7), including anti-BrdU and anti-PH3 antibodies, at 1:100 dilution in blocking buffer overnight at 4°C. For the secondary antibody incubation, a 1:100 dilution in blocking buffer was used, and the samples were incubated for 1 hour at room temperature. Nuclei were counterstained with DAPI. Images from 30 randomly selected high-power fields were taken to evaluate cell proliferation. Cells in the S phase of the cell cycle were identified by analyzing BrdU incorporation and quantified as a percentage of positively stained cardiomyocytes. Likewise, cells in the M phase of the cell cycle were identified by analyzing the expression of PH3 and quantified as the percentage of positively stained cardiomyocytes.

### miRNA overexpression and siRNA-based knocking down.

The hiPSC-CMs were seeded either in 6-well plates (for qPCR analysis, with 2,000,000 cells per well) or in 2-well chamber slides (for histology studies, 500,000 cells per well). The cells were then incubated in medium (RPMI 1640 medium containing 2% B27 supplement) at 37°C for 48 hours. The transfection mixture was prepared by adding transfection reagents (Lipofectamine RNAiMAX) to the fresh culture medium along with either miRNA mimics or siRNAs targeting specific miRNAs (see sequences in Supplemental Table 10). Different concentrations of mimics or siRNAs (1, 5, 10, 20, 100 nM) were used. Cells that were not treated with miRNA mimic or were treated with negative control siRNAs were used as the control group, respectively. On the day of siRNA transfection, the cells were washed with PBS and then treated with the transfection mixture. Both the transfection reagent and siRNAs at varying concentrations were diluted 1:1 in serum-free Opti-MEM and incubated for 5 minutes at room temperature before being added to the cells. Functional experiments (qPCR analysis and histological studies) were carried out 3 days after the siRNA treatments. The effectiveness of miRNA overexpression and siRNA-based miRNA knockdown was assessed by qRT-PCR using primers listed in Supplemental Table 11.

### Evaluation of circRNA intracellular localization by immunostaining.

The hiPSC-CMs were plated in 2-well chamber slides (~500,000 cells per well for RNA probe studies). The cells were then incubated in RPMI 1640 medium containing 2% B27 supplement at 37°C for 48 hours. The samples were fixed with 4% formaldehyde solution at room temperature for 30 minutes, followed by 3 washes with 2 mL of 1× PBS. Samples were then treated with a detergent solution QC for 5 minutes at room temperature, followed by 2 washes with 1× PBS. Next, a working protease solution was prepared and added to the slides, followed by a 10-minute incubation. The working probe set solution was prepared using circRNA probes targeting specific circRNAs designed by Thermo Fisher Scientific. Samples were rinsed with 1× PBS, and then the working probe set solution was applied to the samples with 80 μL of working probe set solution for each sample. This solution contains 0.8 μL of probe set and 79.2 μL of probe set diluent buffer. Samples were incubated for 3 hours at 40°C. The slides were washed with a wash buffer 3 times and then treated with preamplifier mix, amplifier mix, and label probe mix solutions in separate steps, each followed by incubation and washing with the wash buffer. Subsequently, samples were treated with a working DAPI solution, incubated, and washed with 1× PBS. Finally, samples were prepared for microscopy by adding Prolong Gold Antifade Reagent and cover glass, before being imaged on an Olympus IX83 microscope at ×40 original magnification with DAPI, GFP, and Alexa Fluor 594 channels.

### TVEM of cell cycle–regulating circRNAs.

To get an overview of how our circRNA expression profile changed across time, we performed TVEM analysis of circRNA expression, focusing on circRNA host genes that were enriched in cell cycle–related pathways. Variables used in the model are defined below. Consider a cell cycle–related pathway *C_1_^i^*. If *G_all_* is the list of all our host genes in our data, then we identify a set of host genes *g_1_, g_2_ …g_n_* ϵ *G_c_^i^* enriched in cell cycle pathway *i* where *G_c_^i^* ⊂ *G_all_*. To identify if a specific cell cycle pathway is time dependent, we use our selected gene sets *G_c_^i^* as our positive label. We then randomly select *n* negative gene sets *G_noncellCycle_*, such that *G_noncellCycle_* ⊂ (*G_all_* – *G_c_^i^*). As such for every *C_1_^i^* with *n* genes, we have *n* × 2 response variable *Y* indicating whether a host gene is in *C_1_^i^* or not. Additionally, for each of these selected 2*n* host genes, we define our explanatory variable *x* as corresponding circRNA expression data. A time variable is also defined based on the postnatal day from which the data came (i.e., time = 1 if sample was from P1, 3 if sample was from P3, and so forth). For each evaluated pathway, we leveraged *tvem* package v(1.4.1) (81) in R along with the above-defined 2*n* *×* 3 matrix to run the TVEM model as per the equation *y_t_* ~ β_0_(*t*) + β_1_(*t*)*x*(*t*).

We then evaluated the coefficients for different cell cycle–related pathways (Supplemental Figure 3).

### Statistics.

Quantile normalization and subsequent data processing were conducted in R software. The statistical analysis was performed using GraphPad Prism 9 software. All data are presented as the mean ± SEM. Statistical comparisons between 2 groups were carried out using 2-tailed Student’s *t* test, with statistical significance defined as *P* < 0.05. For comparisons among multiple groups, 1-way ANOVA with Tukey’s honestly significant difference test was used, and statistical significance was set at *P* < 0.05.

### Study approval.

All animal protocols were approved by the Institutional Animal Care and Use Committee (IACUC) of the Mayo Clinic. All animal surgical procedures and euthanasia were performed based on approved IACUC protocol and in accordance with the NIH *Guide for the Care and Use of Laboratory Animals* (National Academies Press, 2011).

### Data availability.

All data associated with this study are present in the paper or the supplemental information, and raw data are included in the Supporting Data Values file. Reagents and materials associated with this study are available from the corresponding authors. RNA-sequencing data have been deposited to a public data set repository (NCBI GEO; access ID: GSE269522).

## Author contributions

LT initiated the project, conducted experiments, acquired and analyzed data, and wrote the manuscript. VN analyzed data and wrote the manuscript. Co–first authorship of LT and VN is reflected by their joint intellectual contributions and writing of this manuscript. Order of co–first authorship was determined by relative experimental and data contributions. PL conducted experiments. JW designed research studies and wrote the manuscript. WZ designed research studies, wrote the manuscript, and handled finance.

## Supplementary Material

Supplemental data

Supporting data values

## Version 1 06/25/2024

In-Press Preview

## Version 2 08/08/2024

Electronic publication
