# Supplementary material for: Identification of circular RNAs regulating cardiomyocyte proliferation in neonatal pig hearts
Source: JCI Insight. 2024 Jun 25;9(15):e175625. doi: 10.1172/jci.insight.175625 (PMC11383601; doi:10.1172/jci.insight.175625)
Supplement: Supplemental data [file jciinsight-9-175625-s028.pdf]

Supplemental information

Identification of Circular RNAs Regulating Cardiomyocyte Proliferation in Neonatal Pig Hearts

Ling Tang, Verah Nyarige, Pengsheng Li, Junwen Wang, Wuqiang Zhu

List of Supplemental Items

- Supplemental Figures 1-10
- Supplemental Legends 1-10
- Supplemental Tables 1-11

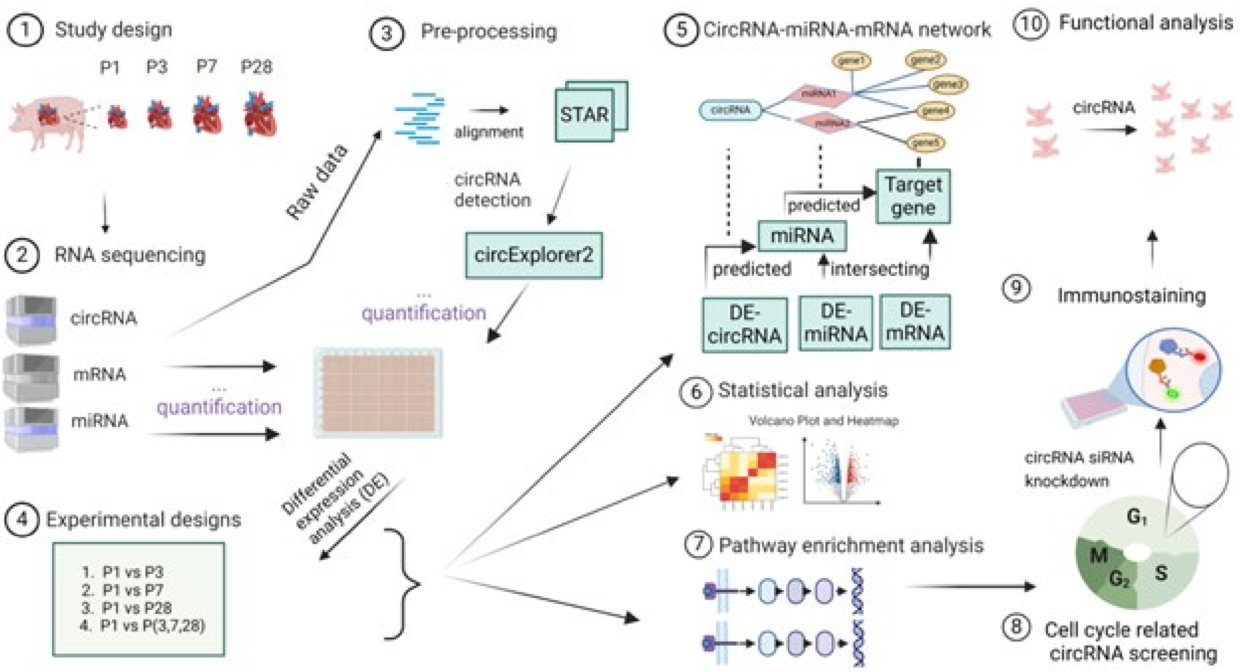

Supplemental Figure 1. Diagram illustrating the experimental design.

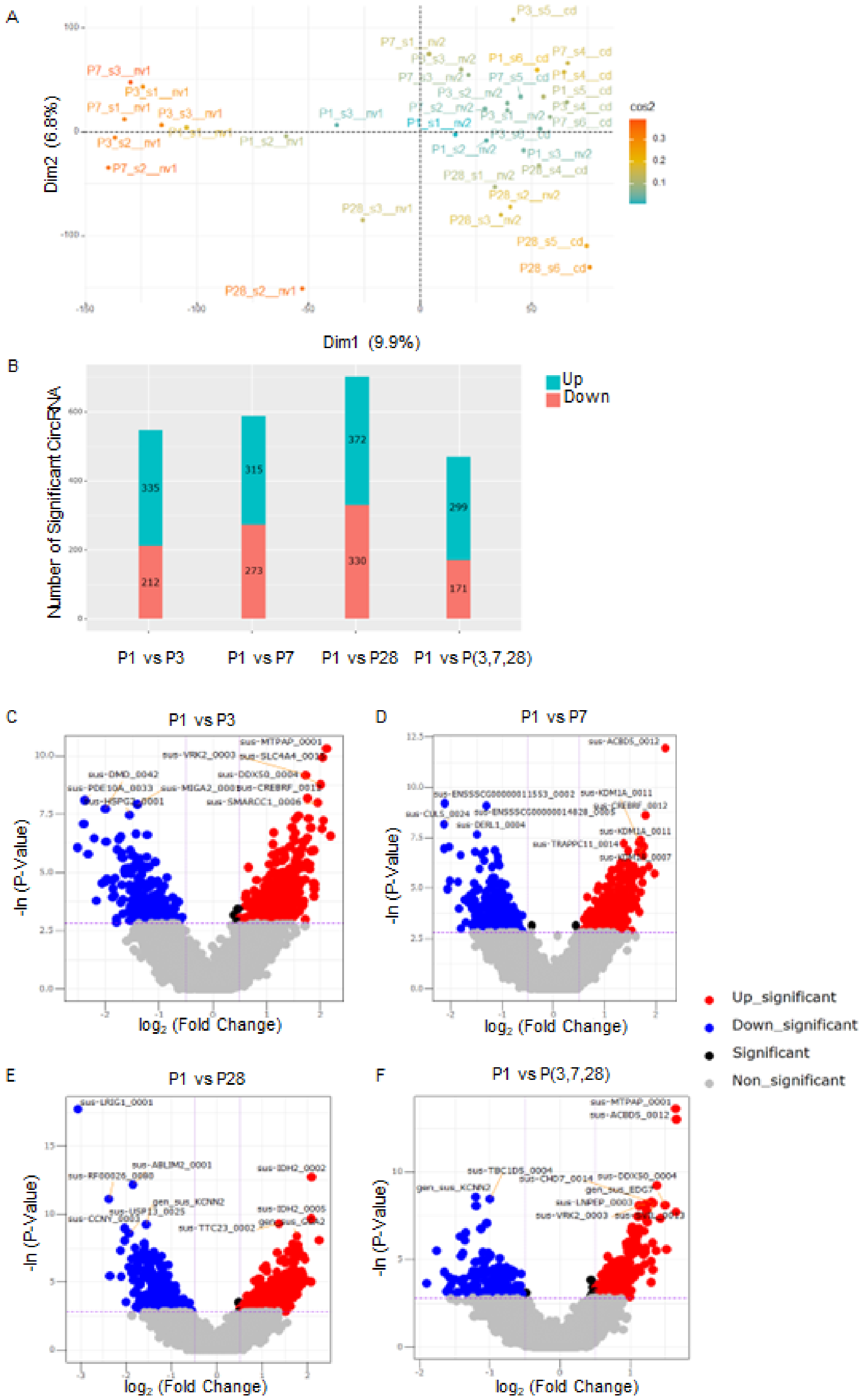

Supplemental Figure 2. Differentially expressed circRNAs in neonatal pig hearts.

(A) Principal Component Analysis (PCA) visualization of batch-corrected circRNA data

from 36 circRNA samples obtained from 24 pig hearts sequenced by CD Genomics Inc. (“cd”), and Novogene Inc. (“nv1” and “nv2”). **(B)** Barplot showing the counts of significantly differentially expressed circRNAs. circRNAs with a $\log_2 FC > 0.5$ and p value < 0.05 were classified as significantly upregulated (in orange) while circRNAs with a $\log_2 FC < -0.5$ and p value < 0.05 were classified as significantly downregulated (in teal). **(C-F)** Volcano plot visualizing differential expression analysis results from various comparisons including P1 versus P3 **(C)**, P1 versus P7 (**D)**, P1 versus P28 **(E**), and P1 versus P3, P7 and P28 **(F)**. circRNAs were categorized as significantly upregulated if they had a p value < 0.05 and $\log_2 FC > 0.5$ (Up_significant in red), significantly downregulated if they had a p value < 0.05 and $\log_2 FC < -0.5$ (Down_significant in blue), significant but neither upregulated nor downregulated if the p value < 0.05 but log2FC < 0.05 (in dark), otherwise nonsignificant (in grey)**.**

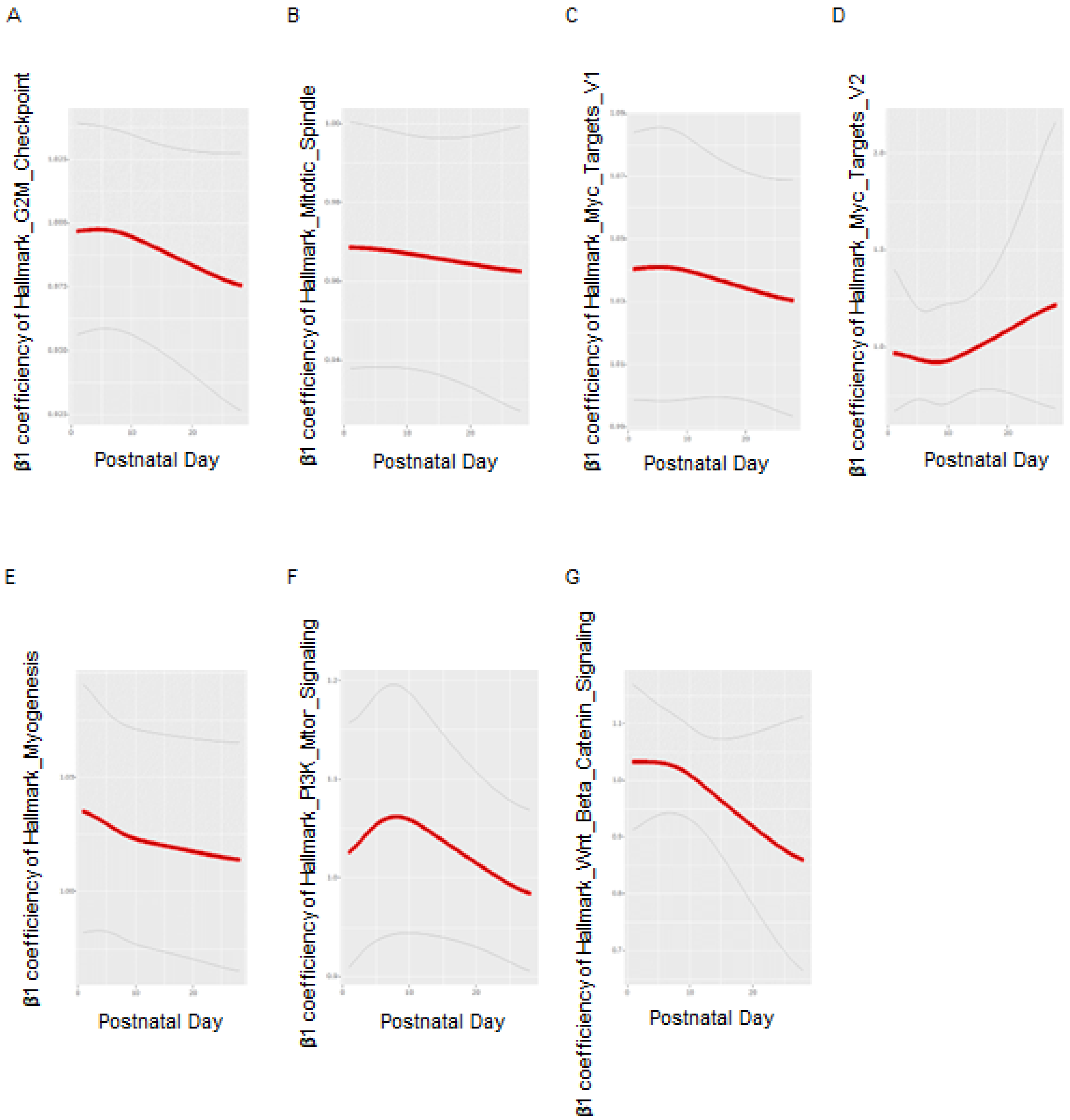

Supplemental Figure 3. Time-varying effect modeling (TVEM) of cell cycle regulating circRNAs. (A-G) Visualization of the changes in the β_1 coefficient across different postnatal periods. Significant variation in the β_1 coefficients over time in core cell cycle related pathways such as G2M checkpoint **(A)**, mitotic spindle **(B)**, Myc-targets **(C-D)**, myogenesis **(E)**, PI3K-Akt-mTOR signaling **(F)**, and Wnt-beta catenin signaling **(G)**.

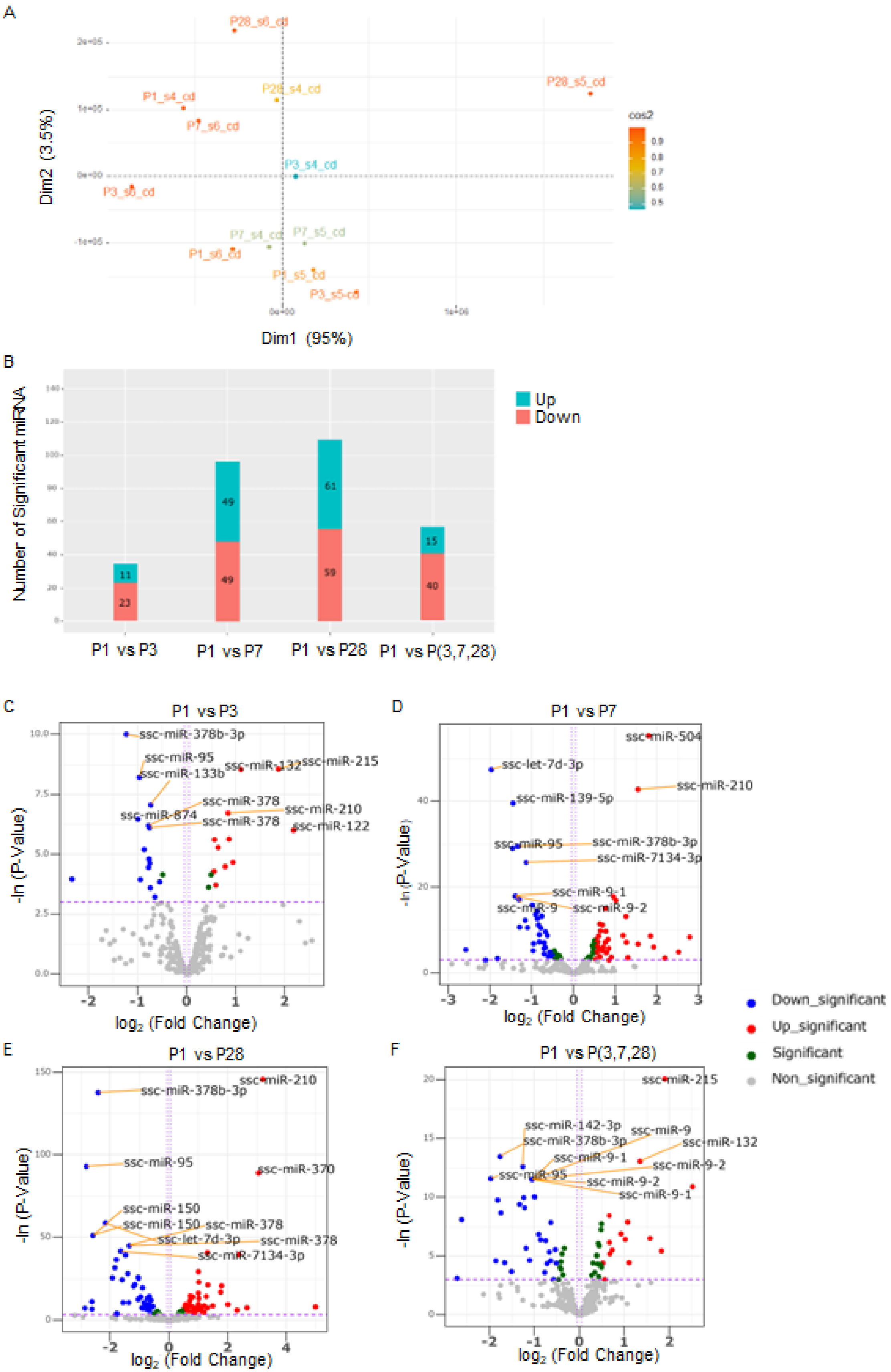

A
Dim2 (3.5%)
Dim1 (95%)
cos2
B
Number of Significant miRNA
Up
Down
P1 vs P3
P1 vs P7
P1 vs P28
P1 vs P(3,7,28)
C
P1 vs P3
D
P1 vs P7
E
P1 vs P28
F
P1 vs P(3,7,28)
-ln (P-Value)
log2 (Fold Change)
Down_significant
Up_significant
Significant
Non_significant

Supplemental Figure 4. Differentially expressed miRNAs in neonatal pig hearts. (A) PCA visualization of 12 miRNA samples obtained from 12 pig hearts at different postnatal days (P1, P3, P7, and P28). **(B)** Barplot showing the counts of significantly differentially expressed miRNAs. MicroRNAs with a $log_2FC > 0.5$ and p value < 0.05 were classified as significantly upregulated (in orange) while circRNAs with a $log_2FC < -0.5$ and p value < 0.05 were classified as significantly downregulated (in teal). **(C-F)** Volcano plot visualizing differential expression analysis of miRNA at different experimental designs including **(C)** P1 versus P3, (**D)** P1 versus P7, **(E**) P1 versus P28, and **(F)** P1 versus P3, P7 and P28. miRNAs were categorized as significantly upregulated if they had a p value < 0.05 and $log_2FC > 0.5$ (Up_signficant distinguished in red), significantly downregulated if they had a p value < 0.05 and $log_2FC < -0.5$ (Down_significant distinguished in blue), significant but neither upregulated nor downregulated if the p value < 0.05 but log2FC < 0.05 (distinguished in green), otherwise nonsignificant (distinguished in grey).

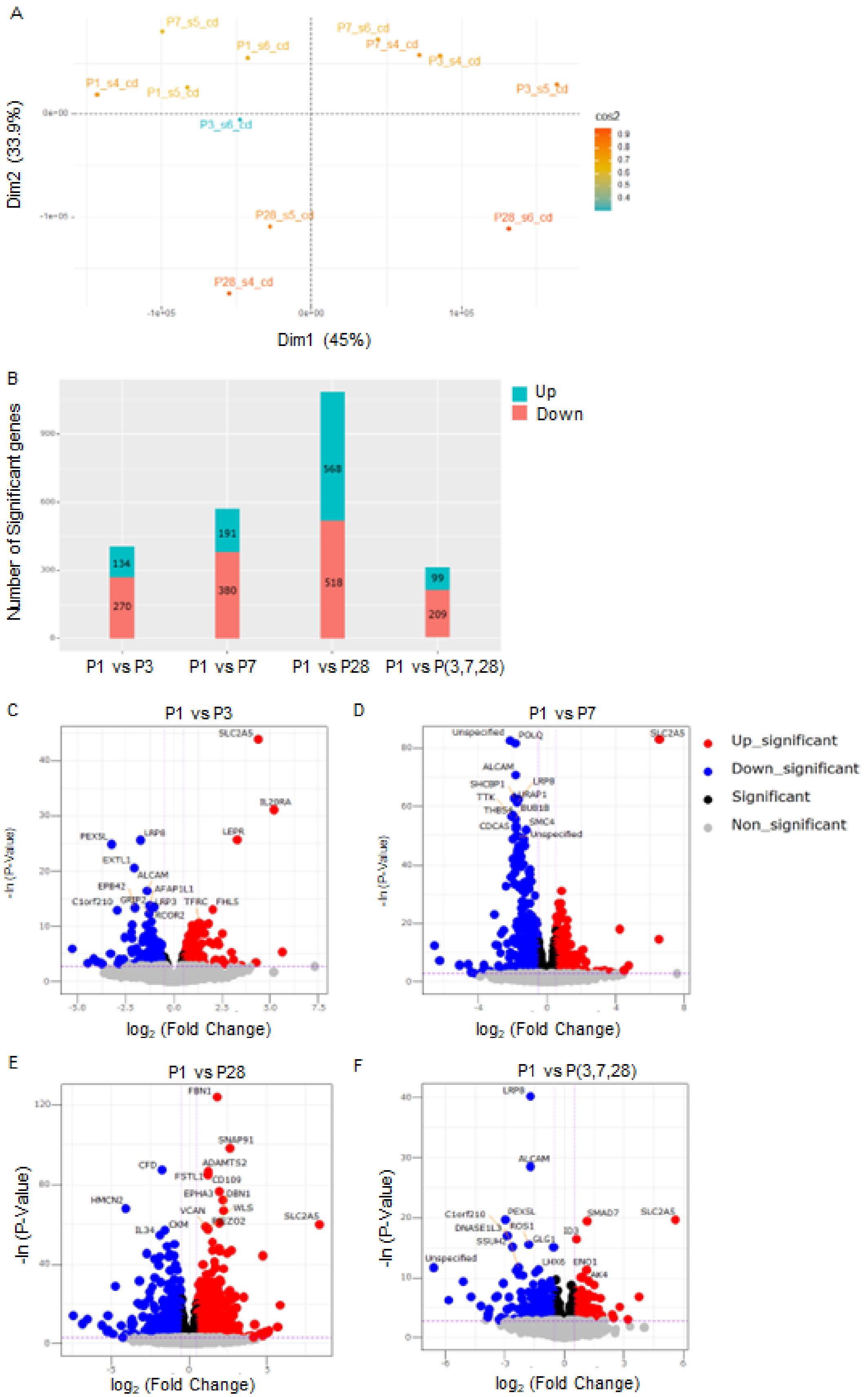
A
Dim2 (33.9%)
Dim1 (45%)
cos2
P7_s5_cd
P1_s6_cd
P7_s6_cd
P7_s4_cd
P3_s4_cd
P1_s4_cd
P1_s5_cd
P3_s5_cd
P3_s6_cd
P28_s5_cd
P28_s6_cd
P28_s4_cd
B
Number of Significant genes
Up
Down
134
270
191
380
568
518
99
209
P1 vs P3
P1 vs P7
P1 vs P28
P1 vs P(3,7,28)
C
P1 vs P3
-ln (P-Value)
log2 (Fold Change)
SLC2A5
IL20RA
LEPR
PEX5L
LRP8
EXTL1
ALCAM
EPB42
AFAP1L1
C1orf210
TFRC
FHL5
D
P1 vs P7
-ln (P-Value)
log2 (Fold Change)
Unspecified
POLQ
SLC2A5
ALCAM
SHCBP1
LRP8
TTK
THBS4
BUB1B
CDCA5
SMC4
Up_significant
Down_significant
Significant
Non_significant
E
P1 vs P28
-ln (P-Value)
log2 (Fold Change)
FBN1
SNAP91
CFD
ADAMTS2
FSTL1
CD109
HMCN2
EPHA3
DBN1
VCAN
WLS
IL34
SLC2A5
F
P1 vs P(3,7,28)
-ln (P-Value)
log2 (Fold Change)
LRP8
ALCAM
C1orf210
PEX5L
SMAD7
SLC2A5
DNASE1L3
ROS1
ID3
Unspecified
LHX6
ENO1

Supplemental Figure 5. Differentially expressed mRNAs in neonatal pig hearts. (A) PCA visualization of 12 mRNA samples obtained from 12 pig hearts at different postnatal days (P1, P3, P7, and P28). **(B)** Barplot showing the counts of significantly differentially expressed mRNAs. mRNAs with a $log_2FC > 0.5$ and p value < 0.05 were classified as significantly upregulated (in orange) while mRNAs with a $log_2FC < -0.5$ and p value < 0.05 were classified as significantly downregulated (in teal). **(C-F)** Volcano plot visualizing differential expression analysis of mRNA from various comparisons including **(C)** P1 versus P3, (**D)** P1 versus P7, (**E**) P1 versus P28, and (**F)** P1 versus P3, P7 and P28. mRNAs were categorized as significantly upregulated if they had a p value < 0.05 and $log_2FC > 0.5$ (Up_signficant in red), significantly downregulated if they had a p value < 0.05 and $log_2FC < -0.5$ (Down_significant in blue), significant but neither upregulated nor downregulated if p value < 0.05 but log2FC < 0.05 (in dark), otherwise nonsignificant (in grey).

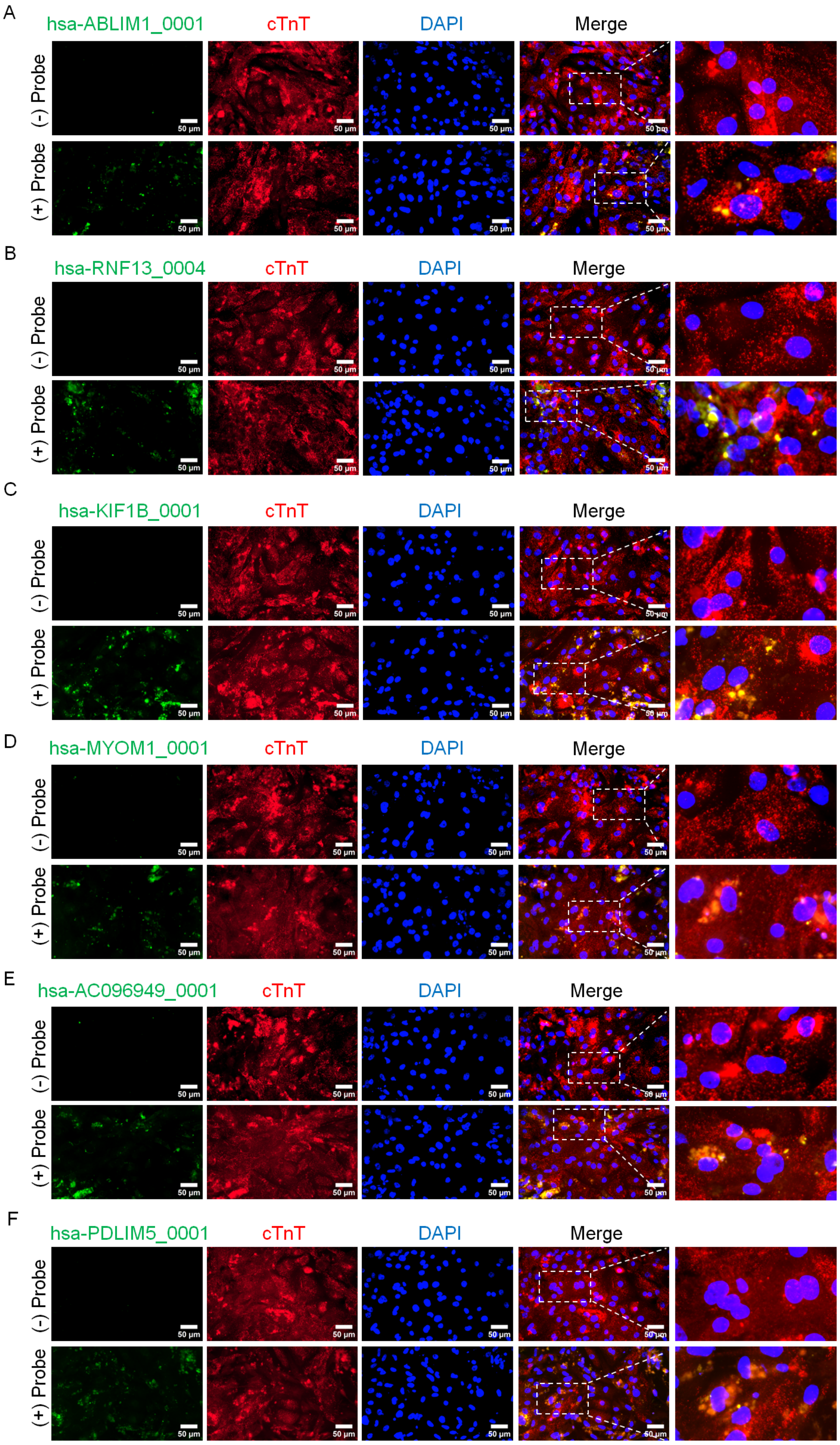
A
hsa-ABLIM1_0001
cTnT
DAPI
Merge
(-) Probe
(+) Probe
B
hsa-RNF13_0004
cTnT
DAPI
Merge
(-) Probe
(+) Probe
C
hsa-KIF1B_0001
cTnT
DAPI
Merge
(-) Probe
(+) Probe
D
hsa-MYOM1_0001
cTnT
DAPI
Merge
(-) Probe
(+) Probe
E
hsa-AC096949_0001
cTnT
DAPI
Merge
(-) Probe
(+) Probe
F
hsa-PDLIM5_0001
cTnT
DAPI
Merge
(-) Probe
(+) Probe
50 μm

1

Supplemental Figure 6. Intracellular localizations of circRNAs. hiPSC-CMs at day 28 after the initiation of cardiac differentiation were utilized. The intracellular location of each circRNA was visualized using an Alexa Fluorescent 488 probe targeting the specific circRNA. Cardiomyocytes were identified using an Alexa Fluorescent 568 probe targeting human cTnT RNA. All cell nuclei were stained with DAPI. Representative images displaying the cytoplasmic and nuclear distribution of hsa-ABLIM1_0001 **(A)**, hsa-RNF13_0004 **(B)**, hsa-KIF1B_0001 **(C)**, hsa-MYOM1_0001 **(D)**, hsa-AC096949_0001 **(E)**, and hsa-PDLIM5_0001 **(F)** in hiPSC-CMs were captured.

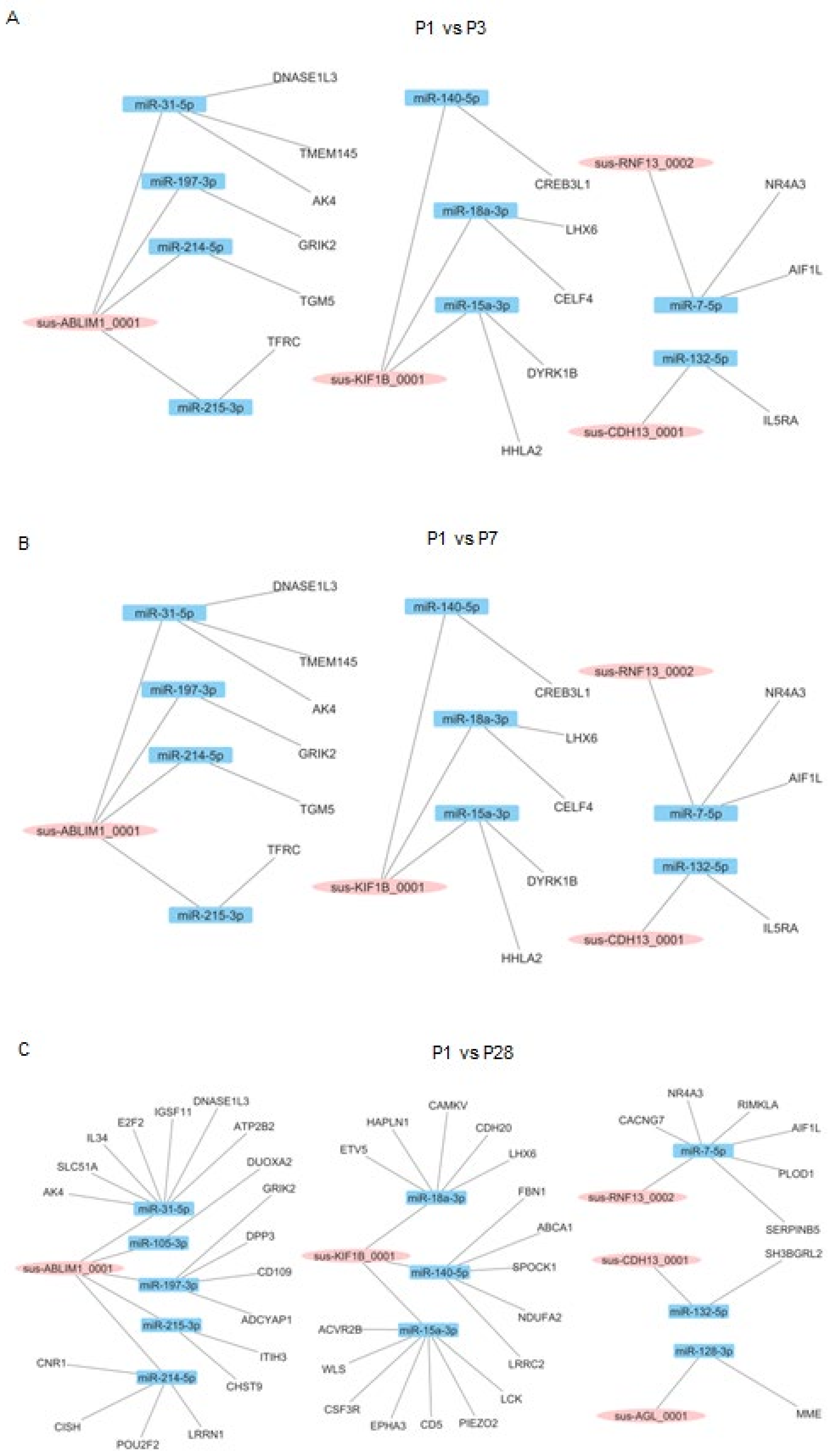

A
P1 vs P3
miR-31-5p
DNASE1L3
TMEM145
AK4
miR-197-3p
GRIK2
miR-214-5p
TGM5
sus-ABLIM1_0001
TFRC
miR-215-3p
miR-140-5p
CREB3L1
miR-18a-3p
LHX6
CELF4
miR-15a-3p
DYRK1B
sus-KIF1B_0001
HHLA2
sus-RNF13_0002
NR4A3
AIF1L
miR-7-5p
miR-132-5p
IL5RA
sus-CDH13_0001
B
P1 vs P7
miR-31-5p
DNASE1L3
TMEM145
AK4
miR-197-3p
GRIK2
miR-214-5p
TGM5
sus-ABLIM1_0001
TFRC
miR-215-3p
miR-140-5p
CREB3L1
miR-18a-3p
LHX6
CELF4
miR-15a-3p
DYRK1B
sus-KIF1B_0001
HHLA2
sus-RNF13_0002
NR4A3
AIF1L
miR-7-5p
miR-132-5p
IL5RA
sus-CDH13_0001
C
P1 vs P28
DNASE1L3
IGSF11
E2F2
IL34
ATP2B2
SLC51A
DUOXA2
AK4
GRIK2
miR-31-5p
miR-105-3p
DPP3
sus-ABLIM1_0001
CD109
miR-197-3p
miR-215-3p
ADCYAP1
ITIH3
CNR1
miR-214-5p
CHST9
CISH
POU2F2
LRRN1
CAMKV
HAPLN1
CDH20
ETV5
LHX6
miR-18a-3p
FBN1
ABCA1
sus-KIF1B_0001
miR-140-5p
SPOCK1
NDUFA2
ACVR2B
miR-15a-3p
WLS
LRRC2
CSF3R
LCK
EPHA3
CD5
PIEZO2
NR4A3
RIMKLA
CACNG7
AIF1L
miR-7-5p
PLOD1
sus-RNF13_0002
SERPINB5
SH3BGRL2
sus-CDH13_0001
miR-132-5p
miR-128-3p
sus-AGL_0001
MME

1

Supplemental Figure 7. Integrated networking analysis of circRNA–miRNA–mRNA interactions. Network diagram illustrates the interactions between circRNAs and miRNAs, as well as miRNA and mRNAs in the comparisons of P1 versus P3 (**A**), P1 versus P7 **(B)**, and P1 versus P28 **(C)**.

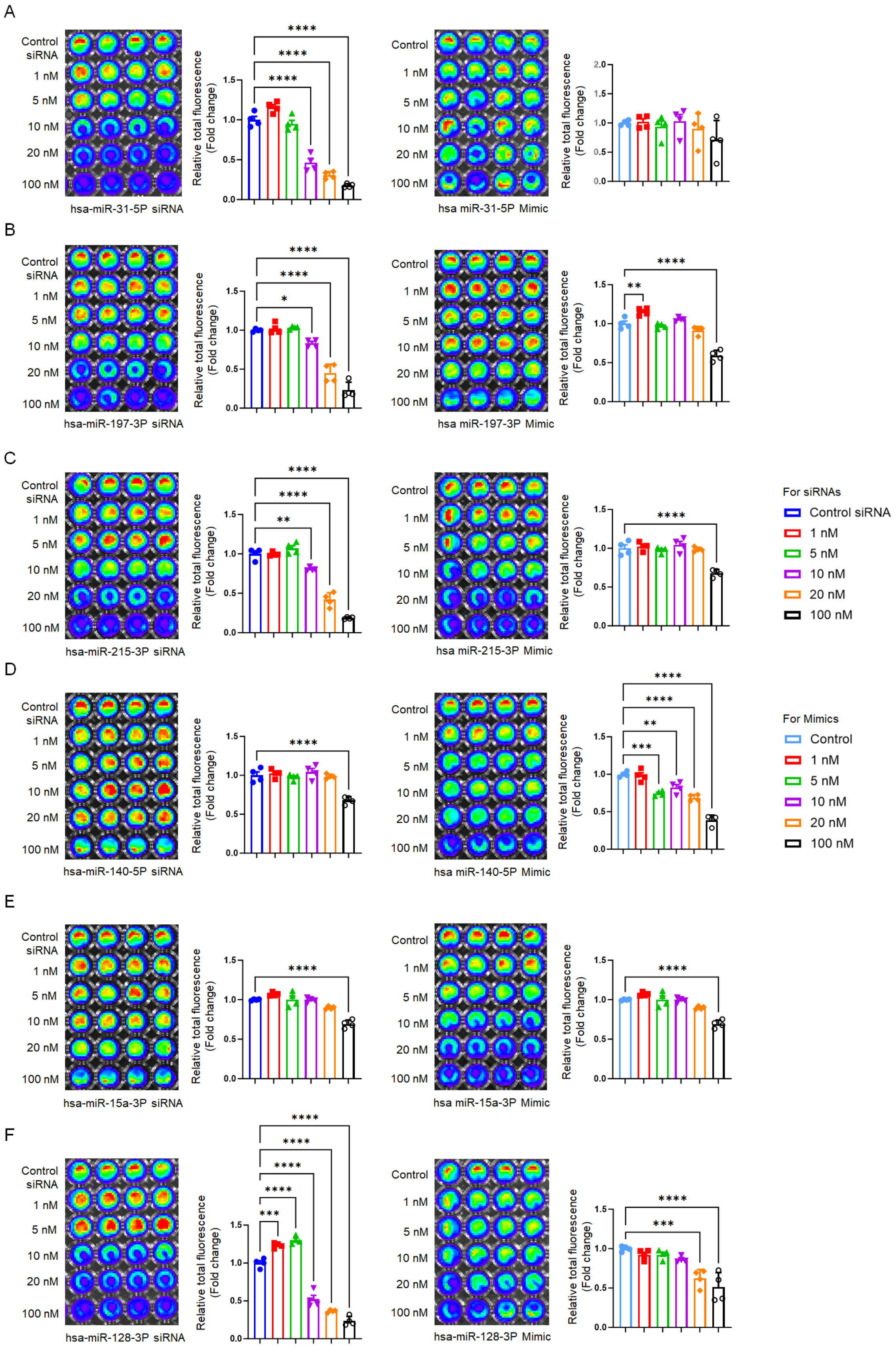
A
B
C
D
E
F
Control siRNA
1 nM
5 nM
10 nM
20 nM
100 nM
Control
hsa-miR-31-5P siRNA
hsa miR-31-5P Mimic
hsa-miR-197-3P siRNA
hsa miR-197-3P Mimic
hsa-miR-215-3P siRNA
hsa miR-215-3P Mimic
hsa-miR-140-5P siRNA
hsa miR-140-5P Mimic
hsa-miR-15a-3P siRNA
hsa miR-15a-3P Mimic
hsa-miR-128-3P siRNA
hsa-miR-128-3P Mimic
Relative total fluorescence (Fold change)
For siRNAs
Control siRNA
1 nM
5 nM
10 nM
20 nM
100 nM
For Mimics
Control
1 nM
5 nM
10 nM
20 nM
100 nM

Supplemental Figure 8. Evaluation of the cell cycle regulatory function of miRNAs. hiPSC-CMs at day 28 after initiation of cardiac differentiation were utilized. The cells were treated with either negative control siRNA, or various concentration (1, 4, 10, 20, and 100nM) of specific human miRNA siRNAs for 3 days. Cell number was measured using bioluminescence analysis for hiPSC-CMs treated with siRNAs or mimics for (A) hsa-miR-128-3p, (B) has-miR-197-3p, (C) has-miR-215-3p, (D) has-miR-140-5p, (E) has-miR-15a-3p, and (F) has-miR-128-3p. All data were presented as mean ± SEM. Statistical analysis was performed via the Student's t-test. n=4 in each group. *p<0.05, **p<0.01, ***p<0.001, and ****p<0.0001.

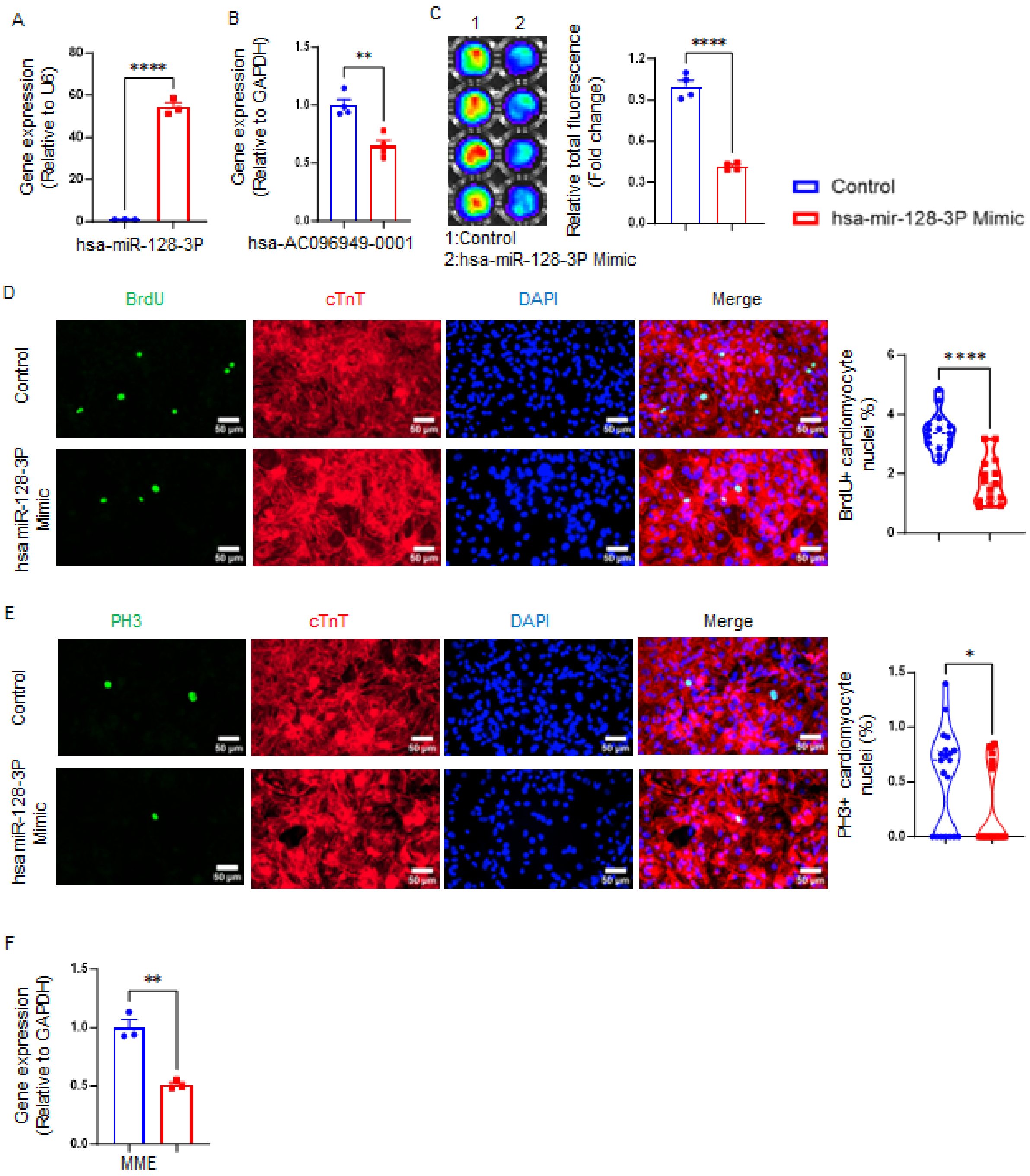

Supplemental Figure 9. Evaluation of the cell cycle regulatory function of has-miR-128-3p mimics. hiPSC-CMs at day 28 after initiation of cardiac differentiation were used. The efficiency of miRNA mimic-based activation in hiPSC-CMs was determined through qRT-PCR. The expression levels of circRNAs were normalized to GAPDH reference gene.

The expression levels of miRNAs were normalized to U6 reference gene. Cell numbers were measured using bioluminescence analysis. Cell cycle activity was determined by immunostaining using antibodies against BrdU and PH3. Cardiomyocytes were identified using anti-human cTnT immunostaining. All cell nuclei were stained with DAPI. The BrdU or PH3 positively stained cardiomyocyte nuclei were normalized to the total number of cardiomyocyte nuclei and the results were presented as a percentage. **(A-B)** Expression of hsa-miR-128-3p **(A)** and its target circRNA hsa-AC096949-0001 **(B)** in the cells treated with has-miR-128-3p mimics was evaluated. **(C-E)** The hsa-miR-128-3p mimics inhibited cell proliferation as indicated by reduced bioluminescence signal **(C)** and the decrease in the prevalence of BrdU- and PH3-positively stained cardiomyocyte nuclei (**D** and **E**). **(F)** hsa-miR-128-3p mimics also inhibited the expression of MME. All data were presented as mean ± SEM. Statistical analysis was performed via the Student's t-test. n=3 technical replicates in each group for panels A and F. n=4 technical replicates in each group for panels B and C. n=15 technical replicates in each group for panel D. n=20 technical replicates in each group for panel E. *p<0.05, **p<0.01, ***p<0.001, and ****p<0.0001.

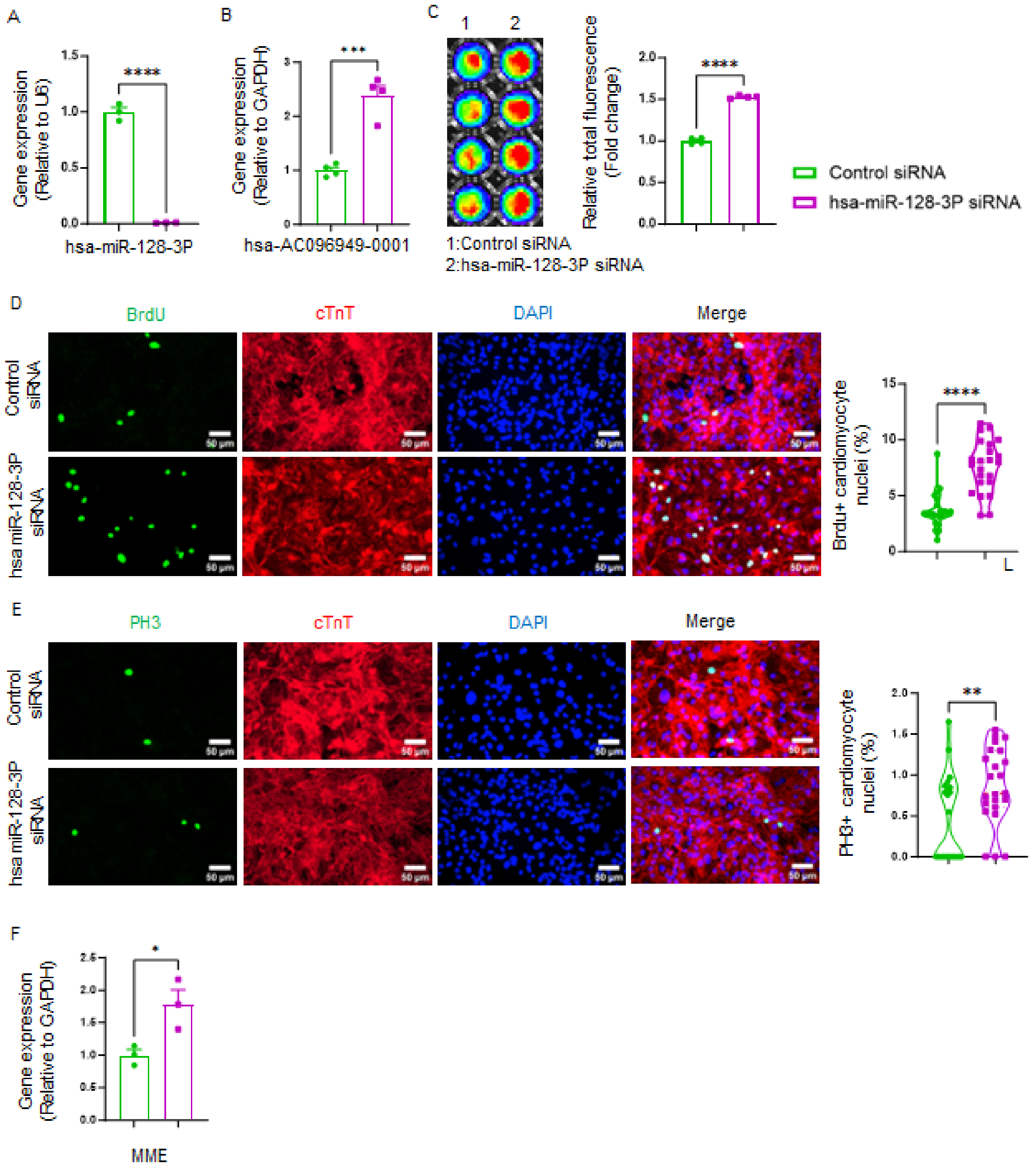

Supplemental Figure 10. Evaluation of the cell cycle regulatory function of has-miR-128-3p siRNAs. hiPSC-CMs at day 28 after initiation of cardiac differentiation were used. The efficiency of siRNA-based knockdown of miRNAs in hiPSC-CMs was determined through qRT-PCR. The expression levels of circRNAs were normalized to

GAPDH reference gene. Cell numbers were measured using bioluminescence analysis. Cell cycle activity was determined by immunostaining using antibodies against BrdU and PH3. Cardiomyocytes were identified using anti-human cTnT immunostaining. All cell nuclei were stained with DAPI. The BrdU or PH3 positively stained cardiomyocyte nuclei were normalized to the total number of cardiomyocyte nuclei and the results were presented as a percentage. **(A-B)** Expression of hsa-miR-128-3p **(A)** and its target circRNA hsa-AC096949-0001 **(B)** in the cells treated with has-miR-128-3p siRNAs was evaluated. **(C-E)** The hsa-miR-128-3p siRNAs promoted cell proliferation as shown by the increase in bioluminescence signal **(C)** and the greater prevalence of BrdU- and PH3-positively stained cardiomyocyte nuclei (**D** and **E**). **(F)** hsa-miR-128-3p siRNAs also enhanced the expression of MME. All data were presented as mean ± SEM. Statistical analysis was performed via the Student's t-test. n=3 technical replicates in each group for panels A and F. n=4 technical replicates in each group for panels B and C. n=23 technical replicates in each group for panels D and E. *$p<0.05$, **$p<0.01$, ***$p<0.001$, and ****$p<0.0001$.

Supplemental table 1. Batch information for RNA sequencing of neonatal pig hearts

Batch ID	Pig ID	Company	RNA sequencing	Data analysis
nv1	P1-s1, P1-s2, P1-s3; P3-s1; P3-s2, P3-s3; P7-s1; P7-s2; P7-s3; P28-s1; P28-s2; P28-s3	Novogene	circRNA only	circRNA (Figure 2)
cd	P1-s4, P1-s5, P1-s6; P3-s4; P3-s5, P3-s6; P7-s4; P7-s5; P7-s6; P28-s4; P28-s5; P28-s6	CD Genomics	Total RNA	circRNA (Figure 2); miRNA (Figure 3); and mRNA (Figure 4)
nv2	P1-s1, P1-s2, P1-s3; P3-s1; P3-s2, P3-s3; P7-s1; P7-s2; P7-s3; P28-s1; P28-s2; P28-s3	Novogene	Total RNA	circRNA (Figure 2)

Supplemental table 2. Hallmark gene sets from pathway enrichment analysis of pig circRNAs

Groups	Pathway	Leading edge genes
P1 vs P3	HALLMARK_MTORC1_SIGNALING	USO1, GGA2, NUFIP1, IFRD1, GSK3B, GSR, PGM1, CANX, GBE1, FADS1, MTHFD2L, IDH1
P1 vs P3	HALLMARK_MYOGENESIS	CDH13, SVIL, BAG1, IFRD1, MYOM1, AGL, ABLIM1, MEF2C
P1 vs P3	HALLMARK_MITOTIC_SPINDLE	DST, MYH10, KIF1B, RANBP9, CDC42, TLK1, STAU1, DLGAP5, KIF20B, NF1, OPHN1, ROCK1, RICTOR, DLG1, PDLIM5, KIFAP3
P1 vs P3	HALLMARK_MYC_TARGETS_V1	EIF4G2, XPOT, UBA2, FAM120A, IFRD1, CUL1, NAP1L1, CLNS1A, VDAC1, DHX15, SRPK1, SYNCRIP, CANX
P1 vs P3	HALLMARK_PROTEIN_SECRETION	SEC31A, DST, KIF1B, USO1, GOLGA4, PAM, ARCN1, ATP1A1, ARFIP1
P1 vs P3	HALLMARK_OXIDATIVE_PHOSPHORYLATION	OAT, HADHA, PDHX, CPT1A, NNT, IDH3A, ACADM, IDH2,

		MTX2, VDAC1, ECH1, ATP6V1C1, IDH1, AFG3L2
P1 vs P3	HALLMARK_ADIPOGENESIS	GPHN, BCL2L13, ANGPT1, IDH3A, ACADM, PDCD4, PGM1, DBT, ECH1, GBE1, PPM1B, IDH1
P1 vs P3	HALLMARK_SPERMATOGENESIS	MLLT10, STRBP, BRAF, EZH2, ZPBP
P1 vs P7	HALLMARK_MYOGENESIS	ABLIM1, SVIL, MEF2A, RB1, CDH13, MYOM1, SORBS1, ACTN2, BAG1, DMD, MEF2C
P1 vs P7	HALLMARK_ESTROGEN_RESPONSE_LATE	PDCD4, FKBP5, CHPT1, UNC13B, FDFT1, IDH2, IL6ST, BAG1
P1 vs P7	HALLMARK_MITOTIC_SPINDLE	DLG1, KIF20B, ROCK1, NF1, TLK1, RANBP9, DST, CDC27, CENPF, KIFAP3, PDLIM5, ARFGEF1, ARHGEF12, RAB3GAP1, KIF5B, PALLD, MYH10
P1 vs P7	HALLMARK_UV_RESPONSE_DN	DLG1, VAV2, SCAF8, BCKDHB, ATRN, ATP2C1,

		VLDLR, AKT3, INPP4B, PDLIM5, LAMC1
P1 vs P28	HALLMARK_MITOTIC_SPINDLE	ARHGEF12, RANBP9, ARFGEF1, DST, CDC27, KIFAP3, DLGAP5, ALMS1, TLK1, CDC42BPA, CCNB2, DLG1, KIF1B, PDLIM5, PKD2, RAB3GAP1, ROCK1, LRPPRC, MYO1E, NF1, STAU1, CENPF, CLIP1, MYH10, TOP2A
P1 vs P28	HALLMARK_MYC_TARGETS_V1	SYNCRIP, DDX21, XPO1, DHX15, XPOT, FAM120A, DEK, CCT3, CUL1, CANX
P1 vs P28	HALLMARK_PROTEIN_SECRETION	GOLGA4, ARFGEF1, DST, MAPK1, ARFGAP3, USO1, ARFIP1, PAM, ARFGEF2, KIF1B, STX12, COPB1, SEC31A
P1 vs P28	HALLMARK_MTORC1_SIGNALING	GGA2, GSK3B, USO1, ENO1, FADS1, PGM1, ME1, PDK1, CANX, SSR1, DDX39A, NUFIP1

P1 vs P28	HALLMARK_G2M_CHECKPOINT	SYNCRIP, CDC27, DMD, XPO1, TOP1, CCNB2, CUL5, SLC7A1, CUL1, NUP98, SLC38A1, DDX39A, CENPF, CBX1, TOP2A, RBL1, KIF4A, SS18, EZH2, SQLE, KIF20B, WRN, ABL1
P1 vs P28	HALLMARK_MYOGENESIS	SVIL, DMD, MYH7, MEF2C, MEF2A, ABLIM1, MYOM1, AGL, BAG1, SH3BGR, RB1, APP
P1 vs P28	HALLMARK_ESTROGEN_RESPONSE_LATE	IDH2, FDFT1, PDCD4, UNC13B, MOCS2, FKBP5, BAG1, TOP2A
P1 vs P28	HALLMARK_E2F_TARGETS	SYNCRIP, DLGAP5, XPO1, ATAD2, CCNB2, DEK, PRKDC, DDX39A, PRPS1, PMS2, TOP2A, NAP1L1, KIF4A, RBBP7, EZH2, MELK
P1 vs P28	HALLMARK_EPITHELIAL_MESENCHYMAL_TRANSITION	DST, HTRA1, BMP1
P1 vs P28	HALLMARK_OXIDATIVE_PHOSPHORYLATION	IDH2, ACADM, PDHX, ACAT1, NDUFS2, DLD, MTX2,

		SUCLA2, LRPPRC, IDH3A, HADHA, ATP6V1C1, SUCLG1, OAT, ETFA, ECH1, AFG3L2, RHOT1, ETFDH
P1 vs P28	HALLMARK_HEME_METABOLISM	ARHGEF12, RBM5, CDC27, EIF2AK1, TOP1, ADD1, MOSPD1, UROS, EZH1, DCAF10, SYNJ1, KDM7A, GAPVD1
P1 vs P28	HALLMARK_ADIPOGENESIS	ACADM, PTCD3, PDCD4, ANGPT1, DLD, PGM1, ME1, ARAF, DBT, IDH3A, ACOX1, SUCLG1, BCL2L13, PPM1B, ECH1, HADH
P1 vs P28	HALLMARK_FATTY_ACID_METABOLISM	ACADM, BCKDHB, PPARA, DLD, SUCLA2, ME1, UROS, ACOX1, SUCLG1, ECH1, HADH, ETFDH, RDH11
P1 vs P28	HALLMARK_APICAL_JUNCTION	CNN2, BMP1, NEXN, DLG1, AKT3, ADAM23, CTNNA1, NF1, MYH10, HADH, SYMPK, VAV2

P1 vs P(3,7,28)	HALLMARK_MITOTIC_SPINDLE	DST, RANBP9, TLK1, DLG1, CDC27, ARHGEF12, NF1, KIFAP3, KIF1B, MYH10, KIF20B, CDC42, ARFGEF1, PDLIM5, DLGAP5, STAU1, PKD2, CENPF, ALMS1, ROCK1, RAB3GAP1
P1 vs P(3,7,28)	HALLMARK_MYOGENESIS	SVIL, MYOM1, ABLIM1, MEF2C, BAG1, MEF2A, CDH13, RB1, AGL, SORBS1, APP
P1 vs P(3,7,28)	HALLMARK_PROTEIN_SECRETION	DST, PAM, USO1, GOLGA4, SEC31A, KIF1B, ARCN1, ARFGEF1, OCRL, ARFIP1, GALC
P1 vs P(3,7,28)	HALLMARK_G2M_CHECKPOINT	SYNCRIP, SLC38A1, CDC27, KIF20B, NUP98, SS18, TOP1, RBL1, ARID4A, CUL1, EZH2, WRN, SLC7A1, DDX39A, CENPF, XPO1
P1 vs P(3,7,28)	HALLMARK_MYC_TARGETS_V1	SYNCRIP, FAM120A, DHX15, NAP1L1, CUL1, UBA2, SRPK1,

		XPOT, HDAC2, DDX21, XPO1, DEK, PTGES3, CCT3
P1 vs P(3,7,28)	HALLMARK_APICAL_JUNCTION	DLG1, NF1, VAV2, AKT3, MYH10, SYMPK, CNN2, NEXN, CTNNA1
P1 vs P(3,7,28)	HALLMARK_ADIPOGENESIS	ACADM, PDCD4, ANGPT1, PPM1B, PGM1, IDH3A, BCL2L13, DBT, ME1, DLD, ECH1, SORBS1, GPHN, PTCD3
P1 vs P(3,7,28)	HALLMARK_HEME_METABOLISM	RBM5, MOSPD1, CDC27, ARHGEF12, EIF2AK1, KDM7A, TOP1, GAPVD1, NNT, PICALM, ADD1, CAST, EZH1, BTRC
P1 vs P(3,7,28)	HALLMARK_MTORC1_SIGNALING	GGA2, USO1, NUFIP1, PGM1, MTHFD2L, GSK3B, DDX39A, ME1, VLDLR, PDK1, GSR

1

Supplemental table 3. Hallmark gene sets from pathway enrichment analysis of pig mRNAs

Groups	Pathway	Leading edge genes
P1 vs P3	HALLMARK_INTERFERON_ALPHA_RESPONSE	ISG15, PARP14, IFI44, MX1, IFI44L, UBE2L6, PLSCR1, BST2, TRIM14, B2M, IFI30, PARP9, TENT5A
P1 vs P3	HALLMARK_INTERFERON_GAMMA_RESPONSE	ISG15, PARP14, IFI44, MX1, IFI44L, SOD2, UBE2L6, PLSCR1, BST2, CD86, MX2, TRIM14, B2M, IFI30
P1 vs P3	HALLMARK_HYPOXIA	SLC2A5, EDN2, CCN2, AK4, TMEM45A, CP, HAS1, ENO1, ADM, ERO1A, RORA, ZNF292, FAM162A, ANXA2
P1 vs P3	HALLMARK_EPITHELIAL_MESENCHYMAL_TRANSITION	PTX3, FSTL3, CCN2, PLOD2, CRLF1, SPOCK1, FBN1, PFN2, LAMA3, FSTL1, TNC, COL12A1, CDH11, TPM1, NT5E, VCAN, COL8A2, PLAUR, TPM4, GADD45A, PLOD1, FBLN2, ABI3BP, FERMT2, GADD45B, EMP3

P1 vs P7	HALLMARK_HYPOXIA	SLC2A5, HAS1, ENO1, ALDOB, PFKL, ISG20, PLAUR, AK4, TMEM45A, BHLHE40, SLC6A6, ERO1A, GAPDHS, PRDX5, RRAGD, FAM162A, ZNF292, NCAN, P3H1, PPP1R15A, ERRFI1
P1 vs P7	HALLMARK_INTERFERON_ALPHA_RESPONSE	ISG15, MX1, ISG20, BST2, IFI30, UBE2L6, PARP14, PARP9, IFI44, BATF2, MVB12A, PLSCR1
P1 vs P7	HALLMARK_OXIDATIVE_PHOSPHORYLATION	NDUFA2, ATP5F1D, COX8A, NDUFB4, LDHB, NDUFS8, TCIRG1, ATP6V0B, ATP5PF, COX4I1, CYB5A, NDUFV1, ATP5PO, ATP5F1A, GPX4, NDUFS3, OPA1, UQCRC1, COX7A2, NDUFV2, TOMM70, TIMM10, NDUFA8, MRPS22, SDHB
P1 vs P7	HALLMARK_INTERFERON_GAMMA_RESPONSE	ISG15, MX1, ISG20, BST2, IFI30, UBE2L6, SOD2, MX2,

		PARP14, IFI44, BATF2, PLSCR1, IRF8
P1 vs P7	HALLMARK_MTORC1_SIGNALING	ENO1, PFKL, TFRC, IFI30, AK4, BHLHE40, SLC6A6, ERO1A, CTH, FADS1, P3H1, FKBP2, SERP1, ME1, TOMM40, HMGCR, PPP1R15A, STIP1, ITGB2, PSMC4, GBE1, HSPA5, PLOD2
P1 vs P7	HALLMARK_G2M_CHECKPOINT	TTK, CDC20, KIF23, NDC80, POLQ, CDKN3, CDKN2C, CCNB2, KIF2C, CHAF1A, RAD54L, INCENP, MCM2, E2F2, STMN1, FBXO5, SMC4, KIF15, SMC2, RPA2, GINS2, STIL, UBE2S, ORC6, HMGN2
P1 vs P7	HALLMARK_E2F_TARGETS	CDC20, BUB1B, CDKN3, DLGAP5, CDKN2C, CCNB2, KIF2C, GINS3, MCM2, MELK, CDCA8, STMN1, DEPDC1, SMC4, WEE1, MMS22L, RPA2, POLD1, PSIP1, UBE2S, LIG1, ORC6, DUT

P1 vs P7	HALLMARK_MITOTIC_SPINDLE	TTK, PIF1, KIF23, NDC80, DLGAP5, CCNB2, KIF2C, INCENP, FBXO5, SMC4, KIF15, ECT2, CDK5RAP2, CKAP5
P1 vs P28	HALLMARK_EPITHELIAL_MESENCHYMAL_TRANSITION	TNC, COL8A2, LAMA1, PTX3, FBN1, SPOCK1, NT5E, CDH11, TPM4, COL12A1, LAMA3, FSTL1, APLP1, PLOD1, OXTR, VCAN, PLOD2, GADD45A, LRRC15, FBLN2, COL5A1, CRLF1, PLAUR, GPX7, THBS2, EMP3, PFN2, TGFBI, PPIB
P1 vs P28	HALLMARK_SPERMATOGENESIS	SLC2A5, SNAP91, ADCYAP1, SCG3, KIF2C, CCNB2, TEKT2, MEP1B, TTK, PHF7, GAPDHS, PCSK4
P1 vs P28	HALLMARK_G2M_CHECKPOINT	KIF15, E2F2, KIF23, CHAF1A, SLC7A5, KIF2C, RAD54L, NDC80, CCNB2, MCM2, GINS2, TTK, FBXO5, STIL, UBE2S, POLQ, STMN1, CDC20, SMC2, SNRPD1, MARCKS

P1 vs P28	HALLMARK_HYPOXIA	SLC2A5, HAS1, CP, TMEM45A, LXN, AK4, ENO1, PFKL, ANXA2, EDN2, GAPDHS, NCAN, TPBG, COL5A1, PLAUR, TGFBI, SLC6A6, AKAP12, AMPD3, CA12
P1 vs P28	HALLMARK_MTORC1_SIGNALING	PSAT1, ME1, AK4, ENO1, PFKL, SLC7A5, HMGCR, PLOD2, MCM2, FADS1, PRDX1, PSMC4, SLC6A6, ARPC5L, TOMM40, TFRC, SORD, SLC1A5, IFI30, DHCR24
P1 vs P28	HALLMARK_E2F_TARGETS	CDCA8, DEPDC1, MELK, DLGAP5, KIF2C, BUB1B, CCNB2, MCM2, MMS22L, UBE2S, STMN1, CTPS1, CDC20, TFRC
P1 vs P28	HALLMARK_OXIDATIVE_PHOSPHORYLATION	COX7A2, NDUFA2, COX8A, SLC25A20, ATP5F1A, ATP5PO, NDUFA8, OPA1, GOT2, ATP6V0B, UQCRC1, ATP5F1D, NDUFB4, TIMM10, MRPL34,

		NDUFV1, ETFB, MRPS15, GPX4, ALAS1, NDUFS3, ATP5PF, NDUFV2, TOMM70, MRPS11
P1 vs P28	HALLMARK_ALLOGRAFT_REJECTION	SIT1, TRAT1, PRKCG, LCK, IL12RB1, CCR2, MAP4K1, CCR5, CD80, NLRP3, CD96, NCR1, CD86, SPI1
P1 vs P28	HALLMARK_INTERFERON_ALPHA_RESPONSE	ISG20, BATF2, IFI44L, TENT5A, UBA7, IFI44, MX1, ISG15, PARP9, PARP14
P1 vs P28	HALLMARK_INTERFERON_GAMMA_RESPONSE	ISG20, BATF2, IFI44L, NLRC5, IFI44, MX1, ISG15, PARP14, CD274, CD86, EIF4E3, B2M, UBE2L6, PDE4B, TNFAIP3, TRIM14, MX2
P1 vs P28	HALLMARK_INFLAMMATORY_RESPONSE	CXCR6, TNFSF15, LCK, ROS1, RASGRP1, CSF3R, C5AR1, ABCA1, PTAFR, NLRP3
P1 vs P(3,7,28)	HALLMARK_EPITHELIAL_MESENCHYMAL_TRANSITION	PTX3, TNC, SPOCK1, COL8A2, CDH11, COL12A1, PLOD2, PLAUR, PFN2, PLOD1, FBN1, VCAN, LAMA3, TPM4, APLP1,

		NT5E, FSTL1, EMP3, GADD45A, PPIB, FSTL3, TPM1, FBLN2
P1 vs P(3,7,28)	HALLMARK_OXIDATIVE_PHOSPHORYLATION	NDUFA2, COX7A2, COX8A, NDUFB4, ATP5F1A, ATP5PO, ATP5F1D, OPA1, ATP5PF, ATP6V0B, NDUFA8, SLC25A20, CYB5A, GPX4, UQCRC1, NDUFV2, MRPL34, TOMM70, NDUFS3, GOT2, NDUFV1, TIMM10, NDUFS8, TCIRG1, MRPS15, COX4I1
P1 vs P(3,7,28)	HALLMARK_HYPOXIA	SLC2A5, HAS1, TMEM45A, ENO1, AK4, CP, PFKL, EDN2, PLAUR, SLC6A6, GAPDHS, FAM162A, ANXA2, ERO1A, ZNF292, RRAGD
P1 vs P(3,7,28)	HALLMARK_MTORC1_SIGNALING	ENO1, AK4, TFRC, ME1, PLOD2, PFKL, IFI30, HMGCR, SLC6A6, ERO1A, PRDX1, PSMC4, FADS1, SERP1, TOMM40, FKBP2, SORD, ETF1, PSAT1, SLC1A5, P3H1, PSMC6, HSPA5

P1 vs P(3,7,28)	HALLMARK_G2M_CHECKPOINT	CDC20, TTK, CDKN3, KIF23, NDC80, INCENP, CDKN2C, POLQ, RAD54L, CCNB2, KIF2C, SLC7A5, CHAF1A, MCM2, E2F2, TGFB1, SMC4, BCL3, STMN1, TRAIP, FBXO5, TLE3, SMC2
P1 vs P(3,7,28)	HALLMARK_E2F_TARGETS	CDC20, GINS3, CDKN3, CDKN2C, BUB1B, CCNB2, WEE1, DLGAP5, KIF2C, PSIP1, MCM2, POLD1, LIG1, SMC4, CDCA8, STMN1, MMS22L, MELK, DEPDC1, SSRP1, RPA2
P1 vs P(3,7,28)	HALLMARK_ALLOGRAFT_REJECTION	SIT1, LCK, TRAT1, PRKCG, ELANE, IL12RB1, CCR2, MAP4K1, CCR5, TGFB1, BCL3
P1 vs P(3,7,28)	HALLMARK_BILE_ACID_METABOLISM	ALDH1A1, LCK, SULT2B1, SLC27A5, LIPE, ABCA1, ALDH8A1, RXRA
P1 vs P(3,7,28)	HALLMARK_ESTROGEN_RESPONSE_EARLY	RASGRP1, SULT2B1, DHRS3, PMAIP1, OLFM1, SLC7A5, CISH, TTC39A, TPD52L1, FAM102A, RPS6KA2, LRIG1,

		CBFA2T3, SEMA3B, SH3BP5, NRIP1, FRK
P1 vs P(3,7,28)	HALLMARK_MITOTIC_SPINDLE	PIF1, TTK, KIF23, NDC80, INCENP, CCNB2, DLGAP5, KIF2C, SMC4, CDK5RAP2, PLEKHG2, SYNPO, FBXO5, ARAP3, MAP1S, EPB41, ABL1, KPTN, TSC1, SPTAN1, ECT2, CYTH2, KIF15, NCK1, CKAP5

Supplemental table 4. Genomic information for validated circRNAs

**Conserved CircRNA ID (Used)**	**Chromosome**	**circAtlas Location**	**Closest circRNA ID (as per our data)**	**circAtlas Location of closest circRNA**	**CD genomic location (Based on our data)**
sus-CDH13_0001	6	5311874-5318531	sus-CDH13_0001	5311874-5318531	5311873-5318531: -
sus-ABLIM1_0001	14	136012794-136046886	sus-ABLIM1_0009	135917668-135932668	135913411-135918906: -
sus-RNF13_0002	13	98121118-98191973	sus-RNF13_0006	98146479-98191973	98162591-98191973:+
sus-KIF1B_0001	6	64542087-64546864	sus-KIF1B_0001	64542087-64546864	64542086-64545302
sus_MYOM1_0001	6	96504635-96512930	sus_MYOM1_0007	96528976-96559830	96538670-96541892:+
sus_TBCD_0003	12	315608-323713	sus_TBCD_0001	289310-301790	301658-323714:+
sus-PDLIM5_0001	8	134379373-134409025	sus-PDLIM5_0008	134355181-134379557	134379372-134409025:-
sus-AGL_0001	4	129654375-129659295	sus-AGL_0386	sus-AGL_VLJW2*	129789460-129792899
sus-ENSSSCG00000026041_0001	No matching sequence in the database				

Supplemental table 5. The match of pig circRNAs to their corresponding human circRNAs

circRNA ID (pig)	circRNA ID (human)
sus-CDH13_0001	None
sus-ABLIM1_0001	hsa-ABLIM1_0001
sus-RNF13_0002	hsa-RNF13_0004
sus-CDC42_0001	None
sus-KIF1B_0001	hsa-KIF1B_0001
sus-MYOM1_0001	hsa-MYOM1_0001
sus-TBCD_0003	None
sus-PDLIM5_0001	hsa-PDLIM5_0001
sus-AGL_0001	hsa-AC096949_0001
sus-ENSSSCG00000026041_0001	None

Supplemental table 6. Key reagents and resources.

Chemicals	Source	Cat. No
B27 supplement	Gibco	17504044
B27 supplement minus insulin	Gibco	A1895601
Bovine Serum Albumin (BSA)	Thermo Fisher Scientific	BP9706100
CHIR99021	BioGems	2520691
D-Luciferin	Sigma-Aldrich	L9504
DMEM	Corning	MT10013CV
DPBS	Gibco	14190250
Dimethyl sulfoxide	Sigma-Aldrich	67-68-5
DAPI	Invitrogen	D1306
High-Capacity RNA-to-cDNA™ Kit	Applied Biosystems™	4387406
iPS DF19-9-7T	WiCells Inc	FTDL-01
IWR-1	BioGems	1128234
KAPA SYBR FAST qPCR Kits	Roche	07959486001
Illumina Ribo-Zero Plus rRNA Depletion Kit	Illumina Inc.	20037135
LightCycler 480 SYBR Green I Master	Roche Inc	04887352001
Lipofectamine™ RNAiMAX	Thermo Fisher Scientific	13778-100
Matrigel	Thermo Fisher Scientific	CB356253
mTeSR™ Plus kit	Stem Cell Technologies	100-0276

MicroRNA first-strand synthesis and miRNA quantitation kit	Takara Bio USA	638316
NEBNext® Ultra™ II Directional RNA Library Prep Kit	New England Biolabs	E7760L
Newborn Calf Serum, New Zealand origin	Thermo Fisher Scientific	16010159
Propidium Iodide - 1.0 mg/mL Solution in Water	Thermo Fisher Scientific	P3566
Qubit™ RNA High Sensitivity (HS) Assay Kits	Thermo Fisher Scientific	Q32852
RPMI 1640	Gibco	11875119
RPMI 1640 with no glucose	Gibco	11879020
RNeasy mini kit	QIAGEN	74104
RIPA Lysis and Extraction Buffer	Thermo Fisher Scientific	89901
Silencer® Select Negative Control #1 siRNA	Thermo Fisher Scientific	4390843
Sodium azide	Sigma Aldrich Inc.	26628-22-8
Two-well Chamber Slide	Thermo Fisher Scientific	154852
TNNT2 RNA Alexa Fluor 568 probe	Thermo Fisher Scientific	PF-210
ViewRNA™ ISH Cell Assay Kit	Thermo Fisher Scientific	QVC0001

Supplemental table 7. Primary and secondary antibodies

Chemicals	Dlutions	Source	Cat. No
Cardiac Troponin T	1:100	Abcam	ab8295
BD Pharmingen™ Alexa Fluor® 647 Mouse Anti-Cardiac Troponin T	1:50	BD Biosciences	565744
BrdU (5-bromo-2'-deoxyuridine)	1:100	Abcam	ab142567
PH3	1:200	Sigma Aldrich Inc.	06-570
Alexa Fluor® 488 AffiniPure Donkey Anti-Rat IgG (H+L)	1:100	JACKSON IMMUNORESEARCH	712-545-150
Alexa Fluor® 488 AffiniPure Donkey Anti-Rabbit IgG (H+L)	1:100	JACKSON IMMUNORESEARCH	711-545-152
Alexa Fluor® 594 AffiniPure Donkey Anti-Rabbit IgG (H+L)	1:100	JACKSON IMMUNORESEARCH	711-585-152
Alexa Fluor® 594 AffiniPure Donkey Anti-Mouse IgG (H+L)	1:100	JACKSON IMMUNORESEARCH	715-585-150

Supplemental table 8. Primer sequences for qRT-PCR based validation of circRNA expression in pig hearts and human induced pluripotent stem cells-derived cardiomyocytes

CircRNA ID	Primer sequences	
sus-CDH13_0001	Forward	AGCATCAGACCATCAGTTTCAAG
	Reverse	TGTGGCGGGATCATCCTTGT
sus-ABLIM1_0001	Forward	AAGAATGAAGTAATGCT TTGAGAAAAGGAAAT
	Reverse	ACATGATTCAGCAATCCCACT
sus-RNF13_0002	Forward	TTCAAAAAAGTATAATTTTGAAAATGCATCTCAG
	Reverse	ACCTTCAGCCGGAAGTCTAT
sus-CDC42_0001	Forward	GATACTGCAGGTCATCATCAAATTTG
	Reverse	ATCGCCCACAACAACACACT
sus-KIF1B_0001	Forward	GCCAAGCCCGCTCTGTGTTG
	Reverse	TGGATGCATTAGCTCCTCCATT
sus-MYOM1_0001	Forward	CGCAAAGCCCGAAACTTCAA
	Reverse	GCGTCAAAATCTACCCCAACAGG
sus-TBCD_0003	Forward	CTTCCCGACTCTCGGACGGC
	Reverse	GGTGGCAGCATGGGGTAGAC
sus-PDLIM5_0001	Forward	ATCTTGAAAGCAGCTAGGGATTGGG
	Reverse	TCTGAGGTCCCAGGCATCAT
sus-AGL_0001	Forward	ATTCTTGGGCCTGTAGTTCTGG
	Reverse	TAAGCATAGCACTGGCGTGT
sus-ENSSSCG	Forward	ACCATCAACCATTGTAGCTTAGT

00000026041_0001	Reverse	AGCCCTACTTACAACAGCCAG
GAPDH-pig	Forward	GTGAACGGATTTGGCCGCA
	Reverse	AAGGGGTCATTGATGGCGAC
hsa-ABLIM1_0001	Forward	AGAAACCACCTTCTCCAGCATG
	Reverse	AGGCTCCCCACATTTATGGC
hsa-RNF13_0004	Forward	GTACATAAATTCAAGAAAGTATAACTTTGAAAATG
	Reverse	GTGGCCCCTTTAAACCTTCAG
hsa-KIF1B_0001	Forward	TGGTTCAAACTTGTGGGGAGGAC
	Reverse	TCCAAGGAAACAGGAAACTTTCGG
hsa-MYOM1_0001	Forward	CCAGAACCTCGTGTCACGTGAC
	Reverse	CTCGGTTTCTTCTAACGTCCTGAG
hsa-	Forward	ATGTTCATCTTGAGAGGTTTTAATATAACTGC
AC096949_0001	Reverse	GTGGCTGGGATTCCTCTGTT
hsa-PDLIM5_0001	Forward	CAACGGCCAAACCAAGGAGCC
	Reverse	CCTTGGACGCCAGTCTTCAGT
MME-human	Forward	GATCTGCTGAGGGGTCACG
MME-human	Reverse	TGTACAAGGCTCAGTGGTGG
GAPDH-human	Forward	GGAGCGAGATCCCTCCAAAAT
	Reverse	GGCTGTTGTCATACTTCTCATGG

Supplemental table 9. Sequences of siRNAs for circRNA knockdown in human induced pluripotent stem cells-derived cardiomyocytes

Genes	Sequences		Concentration (nM)
hsa-	Sense	CUACCAUCCCAAUUGCUUUtt	80
ABLIM1_0001	Antisense	AAAGCAAUUGGGAUGGUAGgt	80
hsa-	Sense	AGAGUAUCACAGACGUGAAtt	10
RNF13_0004	Antisense	UUCACGUCUGUGAUACUCUtt	10
hsa-KIF1B_0001	Sense	AGACCGAAAGUUUCCUGUUtt	10
	Antisense	AACAGGAAACUUUCGGUCUct	10
hsa-	Sense	GGACGUUAGAAGAAACCGAtt	80
MYOM1_0001	Antisense	UCGGUUUCUUCUAACGUCCtg	80
hsa-	Sense	GGUAUUUUCUUCGUGCAAAtt	80
AC096949_0001	Antisense	UUUGCACGAAGAAAAUACCca	80
hsa-	Sense	GAAUCUGAAGCCGAUAAUAtt	40
PDLIM5_0001	Antisense	UAUUAUCGGCUUCAGAUUCtt	40

Supplemental table 10. Sequences of siRNAs for miRNA knockdown and overexpression in human induced pluripotent stem cells-derived cardiomyocytes

**miRNAs**		**Sequences (5'-3')**
hsa-miR-15a-3P	siRNA	GAGGCAGCACAATATGGCCT
hsa-miR-140-5P	siRNA	ACCATAGGGTAAAACCACT
hsa-miR-197-3P	siRNA	CTGGGTGGAGAAGGTGGTGA
hsa-miR-215-3P	siRNA	TGGCCTAAAGAAATGACAGA
hsa-miR-31-5P	siRNA	GCTATGCCAGCATCTTGCC
hsa-miR-128-3P	siRNA	AAAGAGACCGGTTCACTGTG
hsa-miR-15a-3P	Mimic	CAGGCCAUAUUGUGCUGCCUCA
hsa-miR-140-5P	Mimic	CAGUGGUUUUACCCUAUGGUAG
hsa-miR-197-3P	Mimic	UUCACCACCUUCUCCACCCAGC
hsa-miR-215-3P	Mimic	UCUGUCAUUUCUUUAGGCCAAUA
hsa-miR-31-5P	Mimic	AGGCAAGAUGCUGGCAUAGCU
hsa-miR-128-3P	Mimic	UCACAGUGAACCGGUCUCUUU

Supplemental table 11. Primer sequences for qRT-PCR based validation of miRNA expression in human induced pluripotent stem cells-derived cardiomyocytes

CircRNA ID		Primer sequences
hsa-miR-31-5p		5'AGGCAAGAUGCUGGCAUAGCU
hsa-miR-197-3p		5'UUCACCACCUUCUCCACCCAGC
hsa-miR-215-3p		5'UCUGUCAUUUCUUUAGGCCAAUA
hsa-miR-214-5p		5'UGCCUGUCUACACUUGCUGUGC
hsa-miR-105-3p		5'ACGGAUGUUUGAGCAUGUGCUA
hsa-miR-140-5p		5'CAGUGGUUUUACCCUAUGGUAG
hsa-miR-15a-3p		5'CAGGCCAUAUUGUGCUGCCUCA
hsa-miR-128-3p		5'UCACAGUGAACCGGUCUCUUU
U6	Forward	CTCGCTTCGGCAGCACAT
	Reverse	TTTGCGTGTCATCCTTGCG
